# Postbiotics from *Saccharomyces cerevisiae* fermentation stabilize microbiota in rumen liquid digesta during grain-based subacute ruminal acidosis (SARA) in lactating dairy cows

**DOI:** 10.1186/s40104-024-01056-x

**Published:** 2024-08-01

**Authors:** Junfei Guo, Zhengxiao Zhang, Le Luo Guan, Ilkyu Yoon, Jan C. Plaizier, Ehsan Khafipour

**Affiliations:** 1 Department of Animal Science, University of Manitoba, Winnipeg, MB R3T 2N2 Canada; 2 Present Address: College of Food and Biological Engineering, Jimei University, Xiamen, Fujian 361021 China; 3 Department of Agriculture, Food and Nutrition Department, University of Alberta, Edmonton, AB T6G 2R3 Canada; 4 Faculty of Land and Food Systems, University of British Columbia, Vancouver, BC V6T 1Z4 Canada; 5 Diamond V, Cedar Rapids, IA 52404 United States; 6 Present Address: Cargill Animal Nutrition, 15407 McGinty Road West, Wayzata, MN 55391 USA

**Keywords:** Postbiotics, Rumen microbiota, *Saccharomyces cerevisiae* fermentation products, SARA

## Abstract

**Background:**

Subacute ruminal acidosis (SARA) is a common metabolic disorder of high yielding dairy cows, and it is associated with dysbiosis of the rumen and gut microbiome and host inflammation. This study evaluated the impact of two postbiotics from *Saccharomyces cerevisiae* fermentation products (SCFP) on rumen liquid associated microbiota of lactating dairy cows subjected to repeated grain-based SARA challenges. A total of 32 rumen cannulated cows were randomly assigned to 4 treatments from 4 weeks before until 12 weeks after parturition. Treatment groups included a Control diet or diets supplemented with postbiotics (SCFPa, 14 g/d Original XPC; SCFPb-1X, 19 g/d NutriTek; SCFPb-2X, 38 g/d NutriTek, Diamond V, Cedar Rapids, IA, USA). Grain-based SARA challenges were conducted during week 5 (SARA1) and week 8 (SARA2) after parturition by replacing 20% DM of the base total mixed ration (TMR) with pellets containing 50% ground barley and 50% ground wheat. Total DNA from rumen liquid samples was subjected to V3–V4 16S rRNA gene amplicon sequencing. Characteristics of rumen microbiota were compared among treatments and SARA stages.

**Results:**

Both SARA challenges reduced the diversity and richness of rumen liquid microbiota, altered the overall composition (β-diversity), and its predicted functionality including carbohydrates and amino acids metabolic pathways. The SARA challenges also reduced the number of significant associations among different taxa, number of hub taxa and their composition in the microbial co-occurrence networks. Supplementation with SCFP postbiotics, in particular SCFPb-2X, enhanced the robustness of the rumen microbiota. The SCFP supplemented cows had less fluctuation in relative abundances of community members when exposed to SARA challenges. The SCFP supplementation promoted the populations of lactate utilizing and fibrolytic bacteria, including members of Ruminococcaceae and Lachnospiraceae, and also increased the numbers of hub taxa during non-SARA and SARA stages. Supplementation with SCFPb-2X prevented the fluctuations in the abundances of hub taxa that were positively correlated with the acetate concentration, and α- and β-diversity metrics in rumen liquid digesta.

**Conclusions:**

Induction of SARA challenges reduced microbiota richness and diversity and caused fluctuations in major bacterial phyla in rumen liquid microbiota in lactating dairy cows. Supplementation of SCFP postbiotics could attenuate adverse effects of SARA on rumen liquid microbiota.

**Supplementary Information:**

The online version contains supplementary material available at 10.1186/s40104-024-01056-x.

## Introduction

Excessive grain feeding in cattle is associated with gut health disorders, such as acute lactic ruminal acidosis and subacute ruminal acidosis (SARA) [1, 2]. These disorders can occur when animals are rapidly transitioned from low-grain high-forage diets to high-grain low-forage diets, as rumen pH reduces due to increased dietary starch digestion and reduced rumen buffering. The etiology of SARA is complex and multifactorial [1, 3], and is partly associated with increased concentrations of volatile fatty acids (VFAs), but not lactate, that reduce the rumen pH between 5.2 and 5.6 for at least 3 h/d [4]. Apart from dietary factors, such as dietary energy, fiber and starch contents, animal characteristics such as robustness of their rumen and hindgut microbiomes, feeding behavior and capacity of rumen epithelium for absorption of VFA affects the severity of SARA [5–9]. Robustness is defined as the capacity of a microbial community to resist changes caused by the dietary, metabolic, or pathogenic stressors. As such, the degree of microbiome robustness is a key determinant of the microbiome-linked pathologies [10]. In the presence of excess dietary starch, insufficient dietary coarse fiber, and a reduced pH, a less robust rumen microbiome rapidly loses its evenness and becomes dominated by a smaller number of taxa [11, 12]. Low rumen pH may cause the decrease of the relative abundance of fibrolytic bacteria and the increase of amylolytic bacteria in the rumen [13, 14]. This can result in rapid accumulation of organic acids such as VFAs that reduce the rumen pH, and the reduction in the digestive tract barrier function. Consequently, increased concentrations of immunogenic compounds in the digestive tract, such as bacterial lipopolysaccharides (LPS) and histidine, and the translocation of these and other toxic compounds from the digestive tract into the systemic circulation can result in initiation and progression of inflammatory responses [7, 15, 16]. It has been reported that repeated SARA challenges cause more severe depression in rumen pH and increase in ruminal LPS concentrations [17–19]. Improved ration formulation (e.g., via optimizing the level of effective neutral detergent fiber; eNDF), introduction of total mixed ration (TMR), and feed bunk management reduced the occurrence of acute lactic ruminal acidosis during the past decades [6, 20, 21]. However, SARA is still highly prevalent and commonly goes unnoticed resulting in reduced milk production and efficiency of production, compromised animal health and development of secondary diseases that lead to high herd culling rates [22].

One strategy for the prevention of SARA might be supplementation with micronutrients that improve the robustness of digestive tract’s microbiomes [10]. These micronutrients can be divided into two categories, those with direct suppressive effects on other microorganisms, such as antibiotics, ionophores and broad-spectrum antimicrobial compounds; and those with direct promotive effects, such as subsets of prebiotics, probiotics and postbiotics [23]. Suppressive micronutrients, such as ionophores, improve the robustness of microbiomes by the suppression of fast-growing Gram-positive bacteria, which allowing slower growing fibrolytic organisms to maintain their populations [13, 23]. This in turn prevents from loss of community evenness [13, 23]. However, suppressive approaches are usually accompanied with major side effects, such as the development of a resistome against suppressor compounds, and reductions in the diversity of carbohydrate active enzymes (CAZyme) in the microbial community [23]. In contrast, the use of promotive micronutrients can promote influential members of microbiome (i.e., keystone and foundation members) which are essential for maintaining microbiome health and productivity resulting in improved rumen health and enhanced animal production and performance. Prebiotics refers to substrates that are selectively utilized by the host microorganisms conferring a health benefit [24]. Probiotics, or direct fed microbials (DFM) are live microorganisms that may produce a range of antibacterial and bacteriostatic compounds, such as organic acids and bacteriocins, and/or can be involved in the production of amino acids, vitamins, and digestive enzymes [24, 25]. When probiotics or DFM administered in adequate amounts, they should confer a health benefit on the host [24, 26]. Postbiotics has drawn more attention in recent years because of their stability and wide range of mode of action. Postbiotics contain microbial cell components (e.g., pili, cell wall components, internal components), microbial metabolites (e.g., those as a result of anabolic activity of microbes such as vitamins, signaling molecules and neurotransmitters) and intermediate and end-products of microbial fermentation (e.g., those as a result of catabolic activity of microbes such as short chain fatty acids) [27, 28]. The use of postbiotics, improves the robustness of microbiome by the provision of metabolites that stimulate a wide range of influential bacteria, including those with fibrolytic activity. This gives influential microorganisms a competitive edge against faster grower species allowing them to maintain their populations within the microbial community [10, 29, 30]. As such, a postbiotic approach can promote the robustness of a microbiome without affecting its resistome and CAZyme capability [10, 23].

A postbiotic from *Saccharomyces cerevisiae* fermentation (SCFP) (Original XPC, Diamond V, Cedar Rapids, IA, USA) was effective in enhancing the robustness of gastrointestinal microbiome in dairy cows subjected to a grain-based SARA challenge [29]. Our study used two SCFPs, XPC and a next generation product NutriTek (Diamond V). NutriTek contains higher concentrations of antioxidants and polyphenol compounds among other metabolites compared to XPC. In our companion manuscripts [31, 32], we showed that NutriTek was more effective in stabilizing the rumen environment and reducing pro-inflammatory status during SARA compared to XPC. Therefore, we hypothesized that: 1) Grain-based SARA reduces ruminal microbial diversity and the predicted functionality of the microbiome in rumen liquid digesta, and this effect is more severe in a second SARA challenge (SARA 2) compared to the first challenge (SARA 1); 2) SCFPs (XPC and NutriTek) reduce the adverse effects of SARA on the composition and predicted functionality of the microbiome in rumen liquid digesta with NutriTek being more effective with a dose dependency. The objectives of this study were to test these hypotheses by comparing the effects of two commercially available SCFPs (XPC and NutriTek, Diamond V) on characteristics of rumen liquid microbiota in Holstein dairy cows subjected to two grain-induced SARA challenges during early to mid-lactation.

## Materials and methods

The protocol used in this study was approved by the University of Manitoba Animal Care Committee (Protocol # F14-038) and followed the guidelines of the Canadian Council for Animal Care (CCAC, 1993).

### Animals, diet and experimental design

As described previously [32], a total of 32 cannulated lactating dairy cows were used in a randomized complete block design with 8 blocks. Cows were blocked based on parity, previous milk yield, and calving date, and were rumen cannulated approximately 12 weeks before their expected parturition. Cows had fully recovered from the surgery before the start of the study, as evaluated by farm veterinarians. Within each block, cows were randomly assigned to 1 of 4 treatments that were applied from 4 weeks before until 12 weeks after parturition and included: 1) no SCFP (Control), 2) 14 g/d Original XPC (SCFPa, Diamond V), 3) 19 g/d NutriTek (SCFPb-1X, Diamond V), and 4) 38 g/d NutriTek (SCFPb-2X, Diamond V). Supplements were mixed with 140, 126, 121, or 102 g/d of ground corn for treatments 1 to 4, respectively, and fed once daily as a top-dress. Cows were fed a prepartum diet containing 38.7% DM neutral detergent fiber (NDF), 15.5% DM crude protein (CP) and 17.6% DM starch from 4 weeks before parturition and were switched to a lactation diet with 34.9% DM NDF, 17.9% DM CP and 18.6% DM starch until 12 weeks after parturition, except for SARA challenge weeks. During week 5 and week 8 post-parturition, two grain-based SARA challenges were induced (SARA1, SARA2) by replacing 20% of the DM of the lactation TMR with pellets containing 50% ground barley and 50% ground wheat, resulting in a SARA induction diet containing 28.2% DM NDF, 17.2% DM CP and 27.9% DM starch. This replacement was completed gradually over 3 d before the start of the SARA1 and SARA2 challenges. Each SARA challenge lasted for 7 d after which cows were returned to lactation TMR to washout the effects of SARA for two weeks. Cows were fed TMR ad libitum once daily at 0900 h, allowing for between 5% and 10% feed refusals. Animals were housed in individual stalls and had free access to fresh water during the whole experiment. Detailed description of feed sample collection and analyses, chemical and nutrient composition of diets, and confirmation of successful SARA induction (rumen pH reduction) were reported previously [31, 32].

### Sample collection

Whole rumen content samples were collected from 5 sites (cranial, caudal, dorsal, caudal ventral, and caudal dorsal) of the rumen through the rumen canula at 6 h after feed delivery, once weekly at weeks −4, −1, 1, 3, 4 (Pre-SARA1), 7 (Post-SARA1), and 10 (Post-SARA2), and 12 (Post-SARA2) relative to calving. During week 5 (SARA1) and 8 (SARA2), rumen samples were collected twice on the 2^nd^ day (SARA1/1, SARA2/1) and 5^th^ day (SARA1/2, SARA2/2) of the SARA challenge. Rumen solid and liquid digesta were separated with a Bodum coffee filter plunger (ODUM AG, Baar, Switzerland) as described in the companion manuscript [32]. One mL of rumen liquid was subsampled, snap frozen in liquid nitrogen and stored at –80 °C for further microbiota analysis.

### DNA extraction

Frozen rumen liquid samples were thawed on ice and centrifuged at 15,000 × *g* for 10 min to collect the pellets. Their DNA was extracted from the sediment using Quick-DNA ZR Fecal/Soil DNA kits (D6010; Zymo Research Corp., Orange, CA, USA) following manufacturer’s procedures. These included a 2 min bead-beating step at 1,750 strokes/min using Geno/Grinder 2010 (SPEX SamplePrep, Metuchen, NJ, USA) for the mechanical disruption of bacterial cells. Obtained DNA was stored at –80 °C in aliquots of 100 ng/µL (stock) of elution buffer. The DNA was quantified using a NanoDrop 2000 spectrophotometer (Thermo Fisher Scientific, Waltham, MA, USA) and samples were normalized to 20 ng/µL for following amplicon generation using PCR and MiSeq Illumina short-read sequencing.

### PCR amplification and construction of sequencing libraries

The PCR was conducted to amplify V3–V4 hypervariable regions of the bacterial 16S rRNA genes using modified F338/R806 primers [33]. Briefly, the forward PCR primer was indexed with 12-base Golay barcodes, allowing for multiplexing of samples. For each sample, PCR reaction was performed in duplicate and contained 3.0 µL of extracted genomic DNA (20 ng/µL), 1.0 µL of each forward and reverse primer (5 µmol/L), 0.5 µL of 20 mg/mL BSA (Thermo Fisher Scientific), 7.0 µL nuclease-free water (Thermo Fisher Scientific), and 12.5 µL of 5 Prime Hot MasterMix (5 Prime Sciences Inc., Gaithersburg, MD, USA). Reactions consisted of an initial denaturing step at 94 °C for 3 min followed by 32 amplification cycles at 94 °C for 30 s, 55 °C for 20 s, and 72 °C for 20 s, with a final extension step at 72 °C for 5 min in an Eppendorf Mastercycler pro (Eppendorf, Hamburg, Germany). Subsequently, amplicon the sequencing library was generated and sequenced using a MiSeq Reagent Kit V3 (600-cycle; Illumina, San Diego, CA, USA) as described previously [33] at the Gut Microbiome and Large Animal Biosecurity Laboratories, Department of Animal Science, University of Manitoba, Winnipeg, MB, Canada.

### Statistical analysis

#### Bioinformatics analyses of microbiota data

The QIIME2 2023.2. [34] pipeline was used to analyze the 16S rRNA gene amplicon sequencing data. Sequencing data were assigned to their respective samples based on barcode sequences and filtered with default parameters in QIIME2. The DADA2 algorithm was used for quality control and the feature table was constructed where sequences were assigned into amplicon sequence variants (ASVs). All sequences shorter than 200 bp, those containing any ambiguous nucleotide bases and/or a homopolymer length greater than 7 bp were removed from the dataset. Phylogenetic trees were built with FastTree method for further comparison among microbial communities. Taxonomy was classified using a pre-trained Naive Bayes classifier and trained on the Silva database (v.138) 99% OTUs. To reduce systematic variation and ensure the compatibility of the species diversity among the samples, the sampling depth was set at maximum 50,000 sequences in alpha-rarefaction.

Prior to performing downstream analyses, the resulting feature table was filtered to remove samples with low sequencing depths (< 6,072 sequences per sample) according to the results of taxonomy classifier and alpha-rarefaction (Additional file 1). Subsequently, community α-diversity [Shannon’s diversity index, Observed Features index, Faith’s Phylogenetic Diversity (PD) index and Pielou’s Evenness index] and β-diversity (Jaccard distance, Bray-Curtis distance, unweighted UniFrac distance, weighted UniFrac distance) metrics were conducted using QIIME2 default scripts at an even depth per sample. Non-metric multidimensional scaling (nMDS) was applied on the resulting Bray-Curtis distance matrices to generate two-dimensional plots using default settings of the PRIMER-E software (v.7.0.17) [35].

#### Statistical analyses of microbiota data

For comparison of α-diversity and the relative abundances of dominate bacterial phylum, a parametric approach was implemented. Firstly, the normality of distributions of residuals was tested using UNIVARIATE procedure of SAS (v.9.4, SAS Institute Inc., Cary, NC, USA) by Shapiro-Wilk’s statistic. If the residuals were not normal, then the data were transformed to the power of lambda to achieve normality. The lambda was calculated by Box-Cox transformation using TRANSREG procedure of SAS (v.9.4). Original and transformed data were then analyzed by the MIXED procedure of SAS using the model:

$${Y}_{ijk}=\mu +{T}_{i}+{S}_{j}+{P}_{k}+{\left(T\times S\right)}_{ij}+{\left(T\times P\right)}_{ik}+{\left(P\times S\right)}_{kj}+{\left(T\times S\times P\right)}_{ijk}+{e}_{ijk}$$

The variable *Y*_*ijk*_ was dependent on *μ* as the average experimental value and fixed effects of treatment *T*_*i*_ (*i *= Control, SCFPa, SCFPb-1X and SCFPb-2X), stage *S*_*j*_ [*j* = week (−4), week (−1), week 1,…, week 12], parity *P*_*k*_ (*k* = 2 or 3+ parity), and interactions (*T* × *S*)_*ij*_, (*T* × *P*)_*ik*_, (*P* × *S*)_*kj*_ and (*T* × *S* × *P*)_*ijk*_. Block was considered a random factor, and stage as repeated measure. The PDIFF option was applied for pairwise comparisons among treatments, parities, stages and their interactions. Comparisons of least square means were conducted using Tukey HSD tests. Contrast comparisons were made between SARA1 and SARA2 stages, as well as Control and SCFP, SCFPa and SCFPb, and SCFPb-1X and SCFPb-2X. If the effect of parity had a *P*-value more than 0.1, parity and its interactions were removed from the model. Results were presented as least square means with their pooled standard errors (SEM). For interpretation of results, the data that was transformed to achieve normality were presented as means from the original data within tables. Significant effects were considered at *P* < 0.05 and tendencies were discussed at 0.05 ≤ *P* < 0.1.

Permutational multivariate analysis of variance (PERMANOVA; implemented in PRIMER-E software v.7.0.17) was used for the comparison of β-diversity metrics [35]. Label permutations (*n* = 9,999) were used in PERMANOVA to estimate the distribution of test statistics under the null hypothesis that within-group UniFrac or Bray-Curtis measures were not significantly different from between-group measures. The fixed and random factors were incorporated into a PERMANOVA model as described for α-diversity. Further to PERMANOVA, permutational multivariate analysis of dispersion (PERMDISP) was performed in PRIMER-E to test the homogeneity of dispersions among treatments and stages [35].

Compositional dynamics of rumen liquid bacterial communities were assessed using the Metagenomic Longitudinal Differential Abundance (MetaLonDA) method with the edgeR package in R [36]. To achieve this, taxonomic profiles for all samples from all cows were integrated into one count table and normalized with cumulative sum scaling (CSS). Pairwise comparisons were made between Control vs. SCFPa, Control vs. SCFPb-1X, and Control vs. SCFPb-2X through the whole experimental period. Within each comparison, the longitudinal profiles were fitted with a negative binomial smoothing spline. The significant time intervals were identified when *P* < 0.05 after multiple testing corrections using Benjamini-Hochberg false discovery rate (FDR) estimation [37]. Data were presented both at the phylum and genus levels.

#### Functional prediction of rumen liquid microbiota

Predicted functions including amino acid, lipid and carbohydrates metabolic pathways of rumen liquid microbiota were assessed using rumen-specific Phylogenetic Investigation of Communities by Reconstruction of Unobserved States (PICRUSt; CowPI) [38] after filtering the feature table (ASV count > 0.005%). The differences between Control and SCFP supplemented groups were analyzed by STAMP (v.2.1.3) using ANOVA test [39]. Significant differences were adjusted by Tukey-Kramer test and then were identified when *P* < 0.05 after multiple testing corrections by Benjamini-Hochberg’s FDR correction [37].

#### Co-occurrence analysis

A correlation network analysis (CoNet) was used to determine microbial association network and the general organization structure of the microbial community [40]. Positive/negative connections indicate co-occurrence/mutual-exclusion relationships among taxa respectively, and hub taxa were identified when they had more than 15 positive/negative connections with other community members [40] as described previously [41, 42]. The degree of connectedness, a measure used to determine the influential capacity of bacterial taxa [43], was explored at the phylum level by dividing the total number of positive and negative edges observed for each phylum by its relative abundance in the community. When analyzing the microbial association network at the genus level, hub taxa were picked when they had > 15 edges (connections) with other members in the community.

Spearman's rank correlation coefficient (rho) was used to explore the relationships between hub taxa and biodiversity (α- and β-diversity metrics), rumen VFAs as well as rumen ammonia concentrations. Rumen VFAs and lactate were reported in companion paper, which were analyzed using gas chromatography (Model 3900 Star; Varian, Walnut Creek, CA, USA) and ammonia-nitrogen was analyzed using a colorimetric assay [31]. Resulting correlation matrices were visualized in heatmaps generated by the Corrplot package of R [44].

## Results

### Alpha- and beta-diversity dynamics of rumen liquid microbiota

**Fig. 1:**
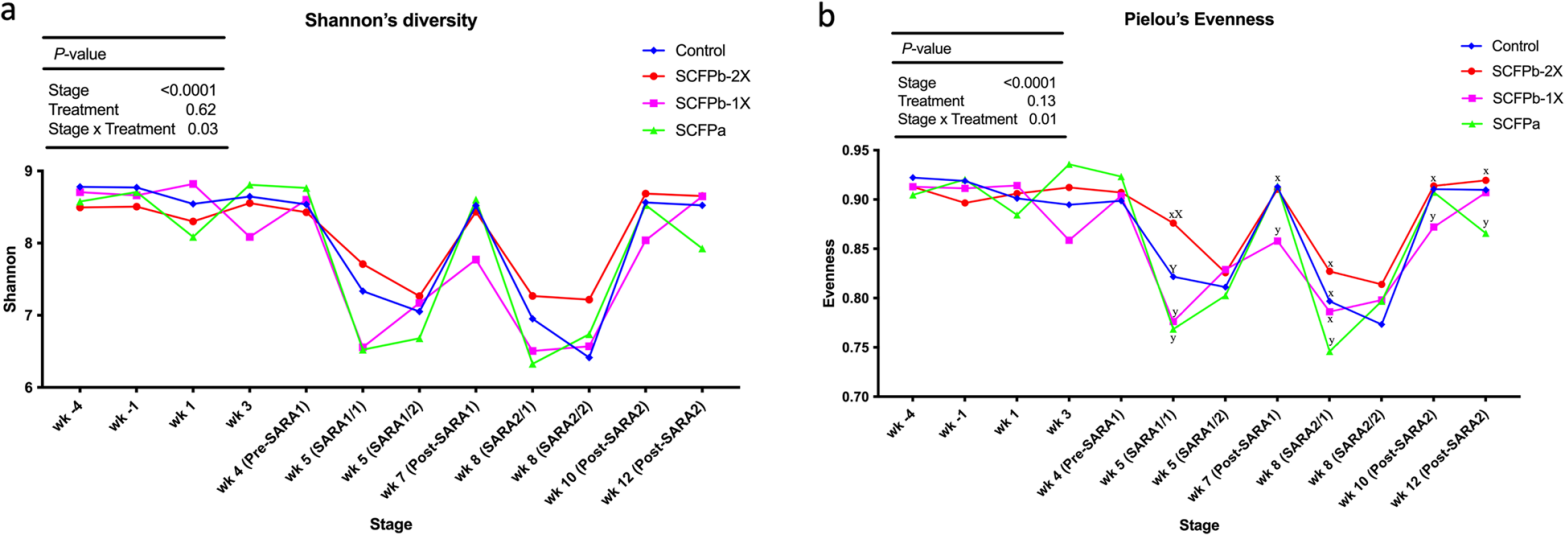
Alpha-diversity dynamics of rumen liquid microbiota. Dynamics of **a**) Shannon diversity index and **b**) Pielou’s evenness index within treatments (Control, SCFPa, SCFPb-1X, and SCFPb-2X) from 4 weeks before until 12 weeks after calving. SARA challenges were conducted on week 5 and week 8 after parturition. Rumen samples were taken weekly but twice during SARA weeks (SARA1/1, SARA1/2, SARA2/1, SARA2/2). Week 4 was considered as Pre-SARA1, week 7 as Post-SARA1, and weeks 10 and 12 as Post-SARA2. x,y means index among treatments are significantly different (*P* < 0.05), and X,Y means index among treatments are tended to be different (0.5 ≤ *P* < 0.1)

**Fig. 2:**
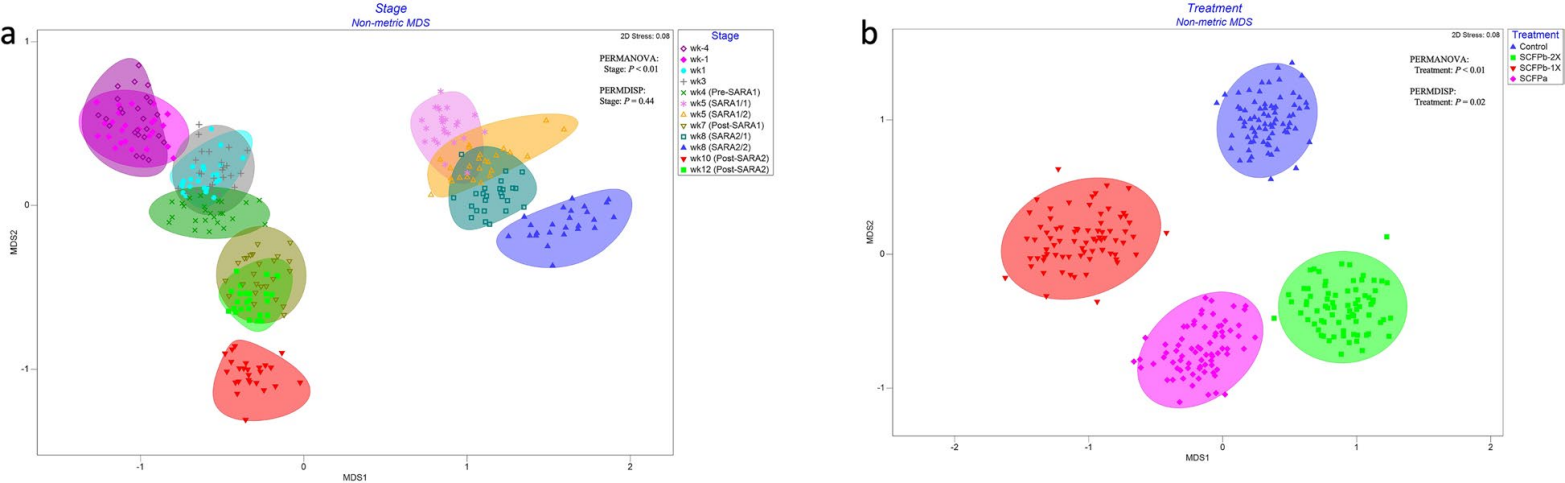
nMDS of Bray-Curtis distances of rumen liquid microbiota. Permutational multivariate analysis of variance (PERMANOVA) was used to detect the distinction of clustering patterns between **a**) stages and **b**) treatments. Homogeneity of dispersions were tested using permutational multivariate analysis of dispersion (PERMDISP). *P* < 0.05 was considered as significant differences. The effect of block was considered as random factor in all comparison. The experimental stage was started from 4 weeks before until 12 weeks after calving. SARA challenges were conducted on week 5 and week 8 after parturition. Rumen samples were taken weekly but twice during SARA weeks (SARA1/1, SARA1/2, SARA2/1, SARA2/2). Week 4 was considered as Pre-SARA1, week 7 as Post-SARA1, and week 10 and 12 as Post-SARA2

There were 26,532 ASVs found in samples and an average of 23,720 ± 6,492 reads per sample through 16S rRNA gene sequencing. The α-diversity of the rumen liquid associated microbiota were reduced (*P* < 0.001) by both SARA challenges across treatment groups (Additional file 2; Fig. 1). There were no differences in these α-diversity indices between the two SARA challenges. Within each experimental stage, evenness, but not richness, was affected by treatments. As showed in Fig. 1B, during the SARA1/1 stage, cows fed SCFPb-2X treatment maintained greater microbial Pielou’s evenness (*P* = 0.01) compared to cows in the SCFPa and SCFPb-1X treatments, tended (*P* = 0.08) to have greater evenness of microbiota in liquid rumen samples compared to the Control cows. The evenness in SCFPb-1X cows was lower than in cows on other SCFP treatments during the Post-SARA1 and Post-SARA2 stages (week 10; *P* < 0.05). During the SARA2/1 stage, the evenness in SCFP-2X cows was greater (*P* = 0.02) than that in SCFPa cows, but not different from those in SCFPb-1X and Control cows. The interaction effect of treatment and stage was significant for Shannon’s diversity index (*P* = 0.03, Fig. 1A), however, no treatment effect was found during each stage. Results from PERMANOVA showed that both treatment (*P* < 0.01, Fig. 2b) and stage (*P* < 0.01, Fig. 2a) affected the β-diversity of rumen liquid microbiota. The PERMDISP results confirmed homogeneity of dispersions among stages (*P* = 0.44; Fig. 2a) but not treatments (*P* = 0.02, Fig. 2b), indicating the treatment effect was influenced by the differences in composition within each group.

### Compositional dynamics of rumen liquid microbiota in response to SARA challenges

**Fig. 3:**
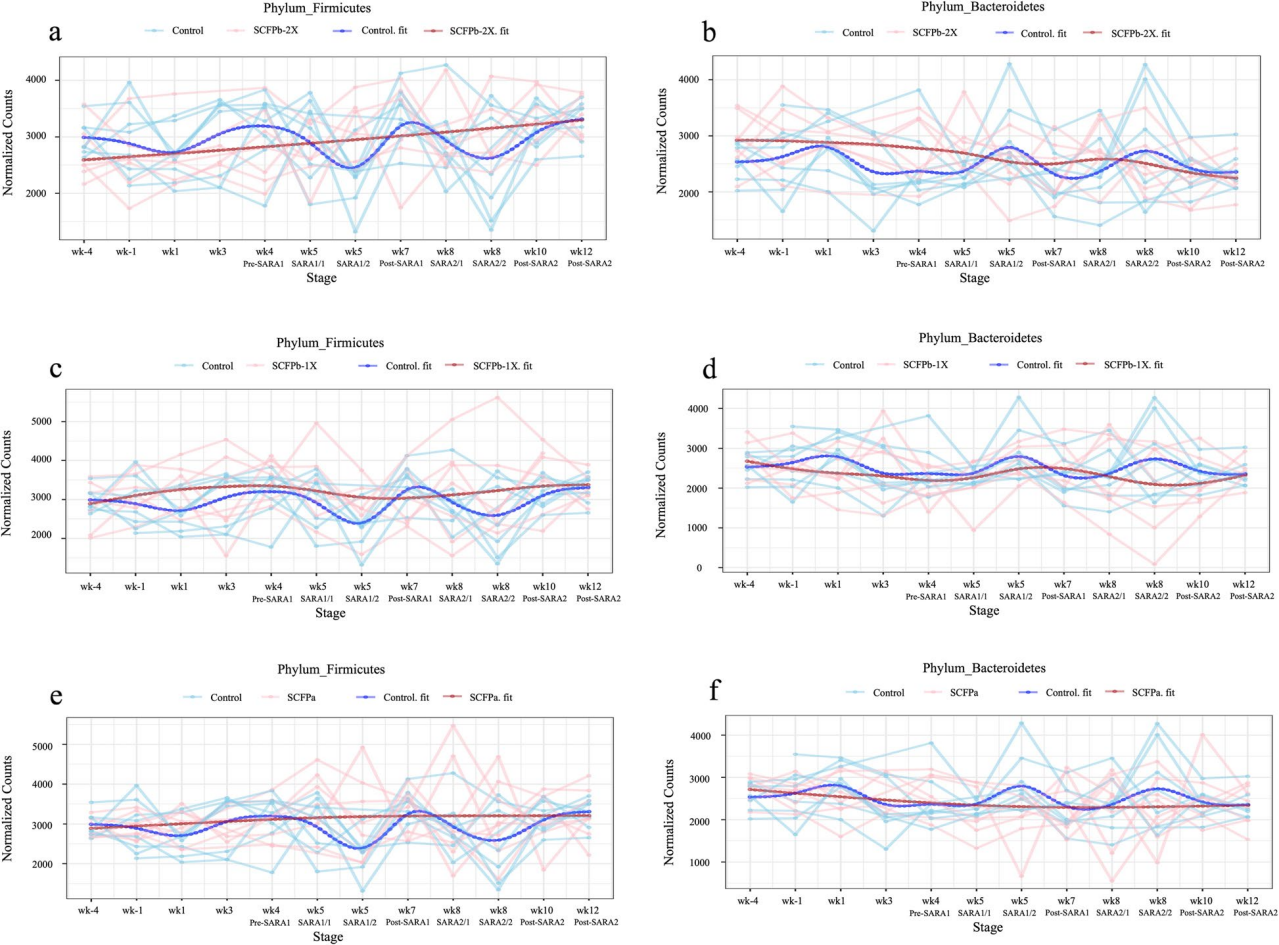
Longitudinal shifts in Firmicutes and Bacteroidetes proportions in rumen liquid microbiota. Metagenomic Longitudinal Differential Abundance (MetaLonDA) was used to test the longitudinal changes in rumen liquid microbiota at the phylum level as lactation progressed. The ASV table was normalized using cumulative sum scaling (CSS) transformation. The longitudinal profiles in each group were fitted with a negative binomial smoothing spline. Blue color represents Control and red represents SCFP groups. The significant time intervals were identified when *P* < 0.05 after multiple testing corrections using Benjamini-Hochberg false discovery rate. **a** and **b** Longitudinal shifts in Firmicutes and Bacteroidetes proportions, respectively, in Control vs. SCFPb-2X group. **c** and **d** Longitudinal shifts in Firmicutes and Bacteroidetes proportions, respectively, in Control vs. SCFPb-1X group. **e** and **f** Longitudinal shifts in Firmicutes and Bacteroidetes proportion, respectively, in Control vs. SCFPa group. Cows received treatments from 4 weeks before until 12 weeks after parturition. SARA challenges were conducted on week 5 and week 8. Rumen samples were collected weekly but twice during SARA weeks (SARA1/1, SARA1/2, SARA2/1, SARA2/2)

In total, 17 phyla and 471 genera were identified. The bacteria in rumen liquid were dominated by members of phyla Firmicutes (48%–52%) and Bacteroidetes (37%–42%), Proteobacteria (3.8%–4.9%), Actinobacteria (0.3%–0.5%), Tenericutes (0.2%–0.4%), and Fibrobacteres (0.1%–0.3%). The average relative abundances of the major bacterial phyla in rumen liquid during non-SARA (Pre-SARA1, Post-SARA1 and Post-SARA2) and SARA (SARA1, SARA2) stages in each treatment group were summarized in Table 1. There was an interaction effect of treatment and stage on the relative abundance of Actinobacteria (*P* = 0.01), and a tended treatment effect on the relative abundance of Fibrobacteres (*P* = 0.05). Both grain-based SARA challenges reduced the relative abundance of Actinobacteria (0.56% vs. 0.32%, *P* < 0.05), Fibrobacteres (0.38% vs. 0.15%, *P* < 0.05) and Tenericutes (0.64% vs. 0.10%, *P* < 0.05), while increased that of Proteobacteria (2.98% vs. 6.67%, *P* < 0.01). The second SARA challenge reduced the relative abundance of Firmicutes compared with Post-SARA2 (47.13% vs. 53.51%, *P* = 0.04), but not with other stages (*P* > 0.05). However, SARA challenges did not influence the relative abundance of Bacteroidetes in rumen liquid microbiota. No treatment effect was observed on the relative abundances of these phyla except for Fibrobacteres, which relative abundance in the SCFPb-1X treatment tended to be lower than in the SCFPb-2X (*P* = 0.07) treatment. The ratio of Firmicutes:Bacteroidetes was not affected by SARA or SCFP supplementation (*P* > 0.05). Longitudinal shifts in proportions of major phyla throughout the experimental period for treatments are provided in Fig. 3 and Additional file 3. Higher fluctuations in the relative abundances of Firmicutes and Bacteroidetes during the first week after parturition and during both SARA challenge stages were observed in the Control cows compared to SCFP supplemented cows (Fig. 3 and Additional file 4).

**Table 1:** Effects of treatment and stage of SARA on the RA^1^ (%) of the bacterial phyla

Item	Treatment^2^				Stage^3^					SEM	*P- *values^4^					
	Control	SCFPa	SCFPb-1X	SCFPb-2X	Pre-SARA1	SARA1	Post-SARA1 (week 7)	SARA2	Post-SARA2 (week 10)		Treat	Stage	Treat × Stage	Contrasts^5^		
														1	2	3
Actinobacteria	0.39	0.44	0.46	0.41	0.56^a^	0.32^b^	0.53^a^	0.32^b^	0.46^ab^	0.04	0.74	**< 0.001**	**0.01**	0.71	0.51	0.42
Bacteroidetes	40.22	38.53	37.45	41.62	40.27	40.15	38.96	39.63	38.27	1.37	0.18	0.86	0.46	0.19	0.27	0.38
Firmicutes	48.52	51.28	51.80	49.08	50.95^ab^	47.48^abB^	52.01^ab^	47.13^b^	53.51^aA^	0.24	0.44	**0.02**	0.58	0.47	0.37	0.23
Proteobacteria	4.81	4.20	4.61	3.88	2.98^b^	6.67^a^	3.55^b^	6.70^a^	3.19^b^	0.39	0.58	**< 0.001**	0.95	0.26	0.53	0.57
Fibrobacteres	0.26^A^	0.27^A^	0.18^B^	0.28^A^	0.38^a^	0.15^b^	0.33^a^	0.13^b^	0.34^a^	0.04	**0.05**	**< 0.001**	0.65	0.21	0.22	0.07
Tenericutes	0.29	0.28	0.21	0.32	0.64^a^	0.10^b^	0.46^a^	0.07^b^	0.57^a^	0.03	0.16	**< 0.001**	0.25	0.14	0.21	0.16
F:B^6^	1.22	1.36	1.37	1.20	1.28	1.20	1.35	1.20	1.41	0.28	0.35	0.36	0.56	0.33	0.13	0.87

^a,b^Means in a row with different superscripts among Treatments are different (*P* < 0.05)

^A,B^Means in a row with different superscripts among Treatments tended to be different (0.05 ≤ *P <* 0.1)

^1^RA: Relative abundances

^2^Treatment: Control = 140 g/d ground corn; SCFPa = 14 g/d Diamond V Original XPC mixed with 126 g/d ground corn; SCFPb-1X = 19 g/d NutriTek mixed with 121 g/d ground corn; SCFPb-2X = 38 g/d NutriTek mixed with 102 g/d ground corn

^3^Stage: SARA was induced during weeks 5 (SARA1) and 8 (SARA2) post-calving. Week 4 was considered as Pre-SARA1, week 7 as Post-SARA1, and week 10 as Post-SARA2

^4^Statistical analyses were conducted on log-transformed data for Actinobacteria and Firmicutes, original data for Bacteroidetes, and Box-cox data for the rest. Presented means are original values prior to transformation

^5^Contrasts: 1 = Control vs. SCFP, 2 = SCFPa vs. SCFPb, 3 = SCFPb-1X vs. SCFPb-2X

^6^F:B: Ratio of Firmicutes:Bacteroidetes

**Fig. 4:**
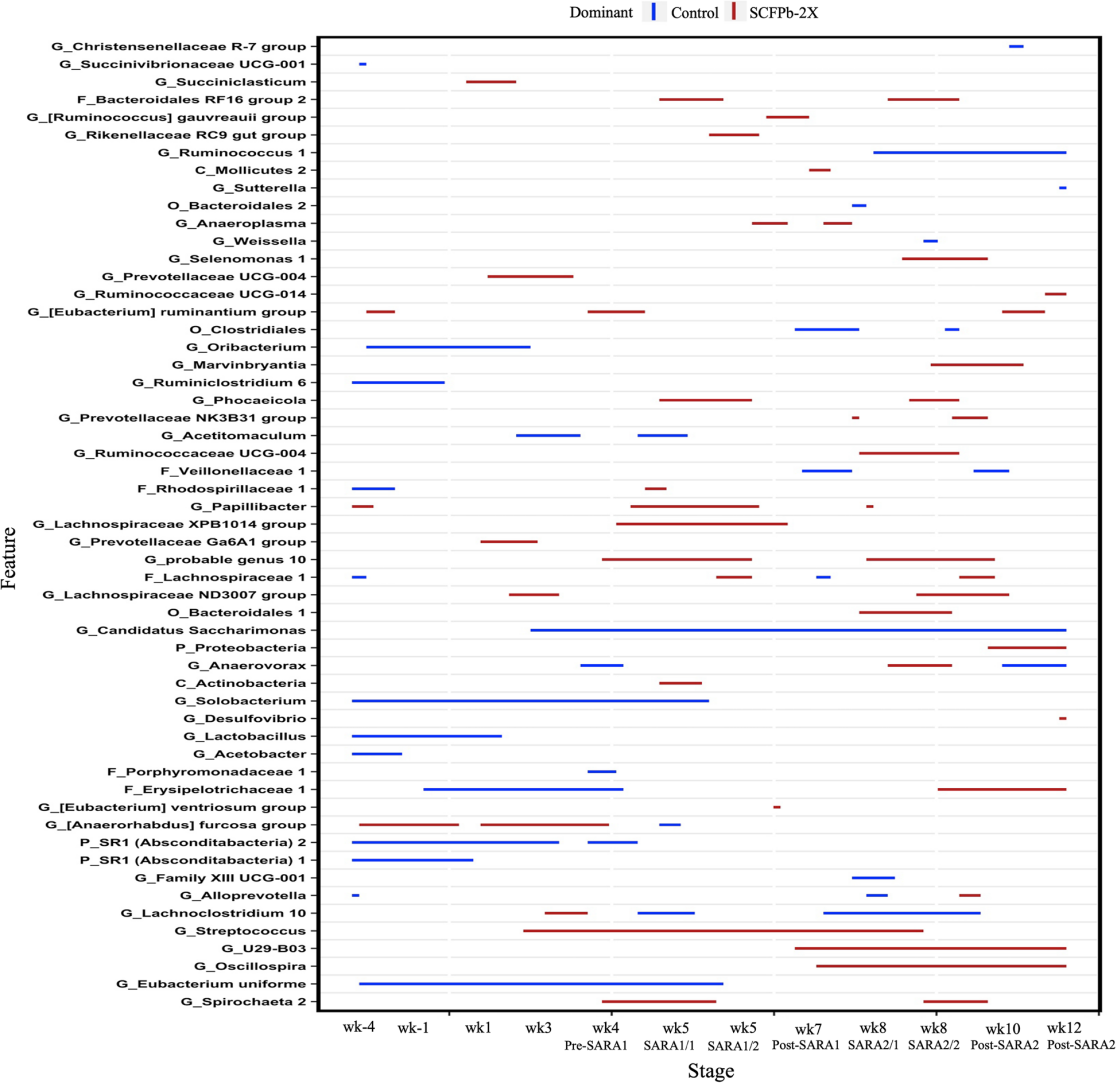
Differentially abundant taxa in rumen liquid microbiota in Control vs. SCFPb-2X. The graph summarizes data that were generated using Metagenomic Longitudinal Differential Abundance (MetaLonDA) method. The significant time intervals were identified when *P* < 0.05 after multiple testing corrections using Benjamini-Hochberg false discovery rate. *X*-axis represents stage relative to parturition. *Y*-axis represents taxa that were promoted by Control (blue lines) and SCFPb-2X (red lines) groups during corresponding stages

**Fig. 5:**
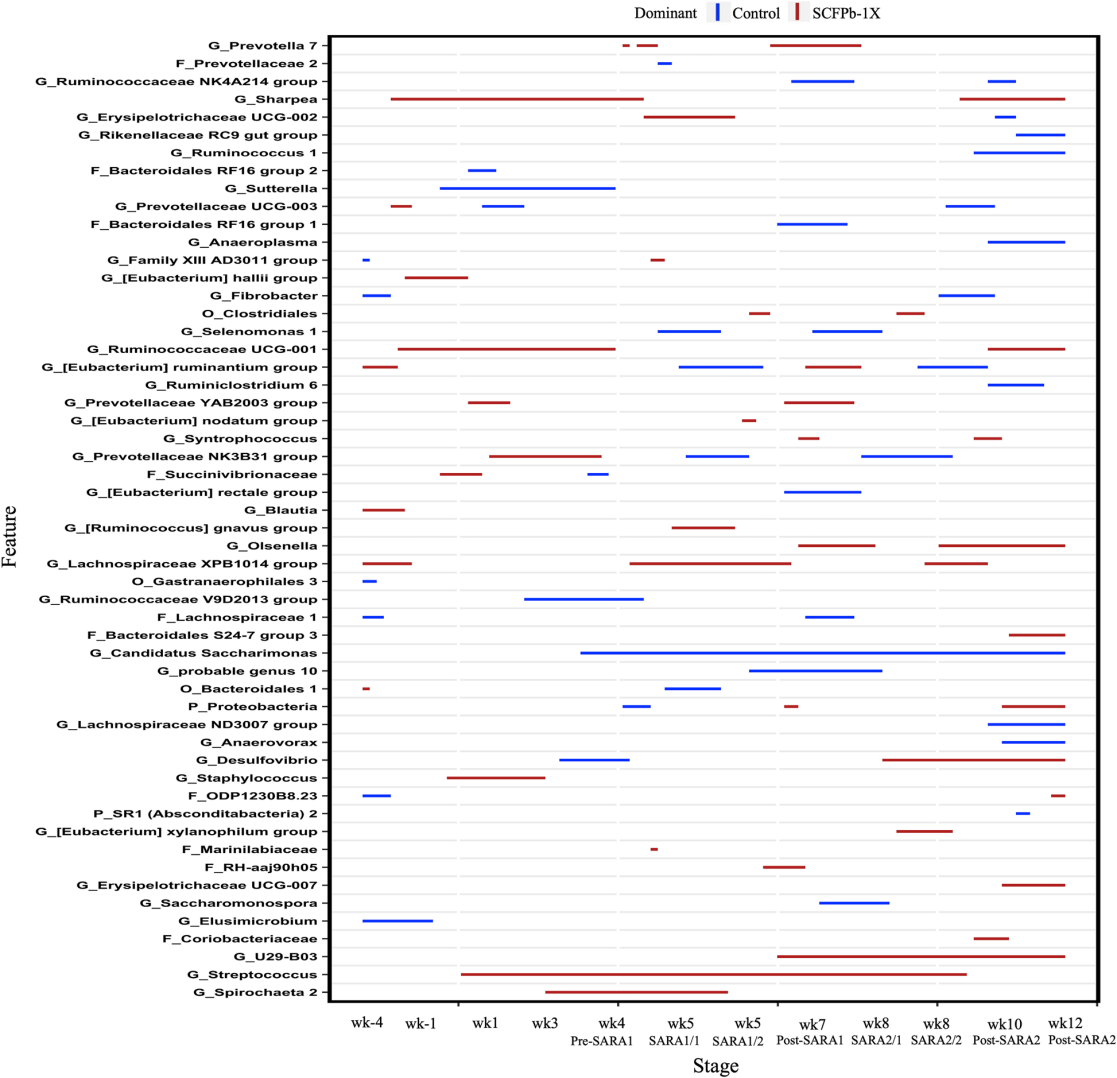
Differentially abundant taxa in rumen liquid microbiota in Control vs. SCFPb-1X. The graph summarizes data that were generated using Metagenomic Longitudinal Differential Abundance (MetaLonDA) method. The significant time intervals were identified when *P* < 0.05 after multiple testing corrections using Benjamini-Hochberg false discovery rate. *X*-axis represents stage relative to parturition. *Y*-axis represents taxa that were promoted by Control (blue lines) and SCFPb-1X (red lines) groups during corresponding stages

**Fig. 6:**
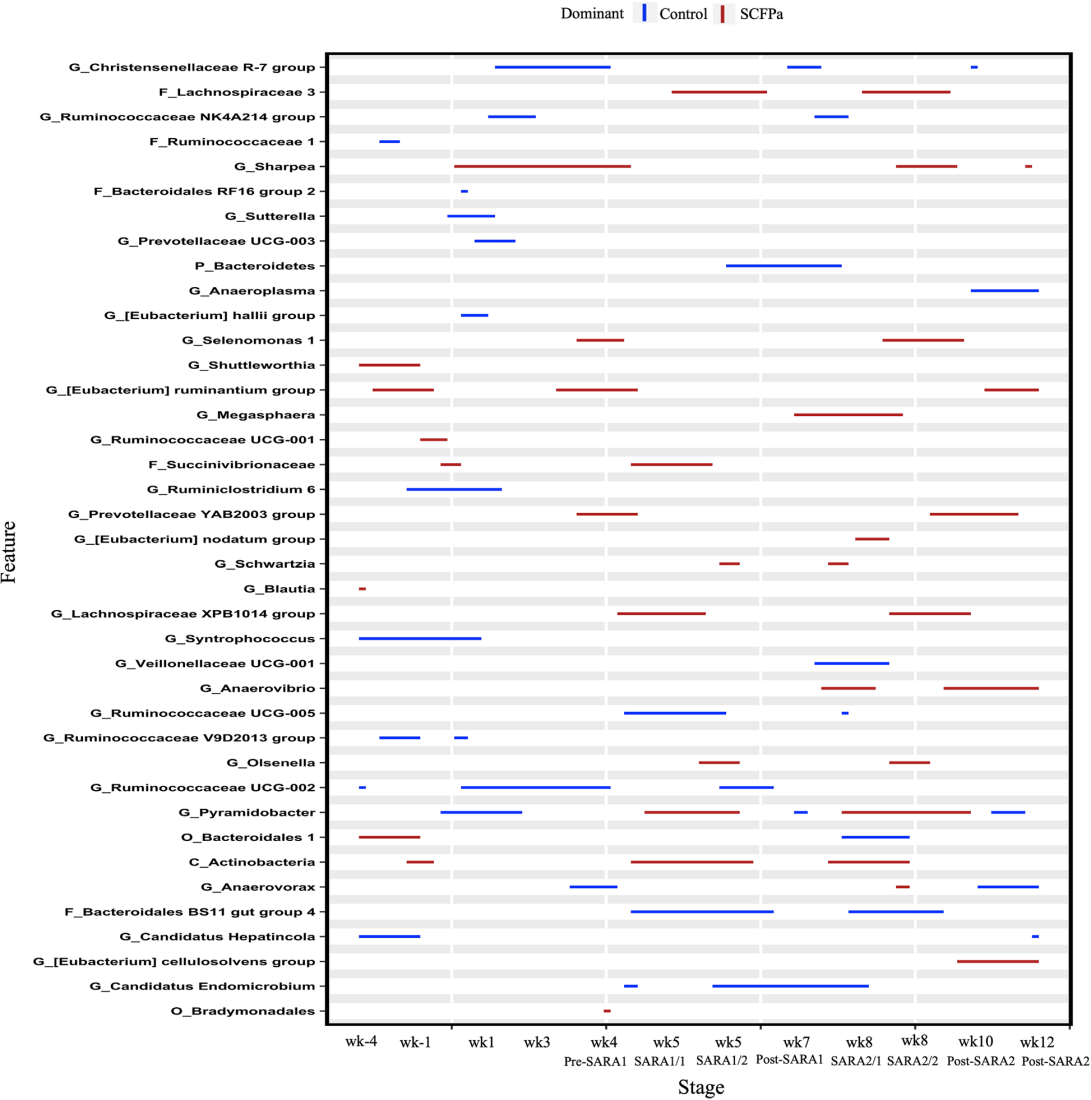
Differentially abundant taxa in rumen liquid microbiota in Control vs. SCFPa. The graph summarizes data that were generated using Metagenomic Longitudinal Differential Abundance (MetaLonDA) method. The significant time intervals were identified when *P* < 0.05 after multiple testing corrections using Benjamini-Hochberg false discovery rate. *X*-axis represents stage relative to parturition. *Y*-axis represents taxa that were promoted by Control (blue lines) and SCFPa (red lines) groups during corresponding stages

The relative abundances of 83 taxa were compared between Control and SCFPb-2X (Fig. 4) treatment groups throughout the 16-week study period. The relative abundances of several taxa, including members of Bacteroidales RF16, Ruminococcaceae, Prevotellaceae, Lachnospiraceae, Rikenellaceae RC9*, Selenomonas* and *Spirochaeta* were higher in SCFPb-2X treatment group compared to the Control treatment during the SARA stages (*P* < 0.05). Similarly, when comparing the Control and SCFPb-1X treatments, the relative abundances of 83 bacterial taxa were higher in one of these 2 treatment groups within corresponding experimental stage (Fig. 5). The relative abundance of several taxa including, that of members of Ruminococcaceae, Prevotellaceae, Lachnospiraceae*, Sharpea, Olsenella*, *U29-B03*, *Streptococcus* and *Spirochaeta* were higher in SCFPb-1X treatment compared to the Control treatment during SARA challenges (*P* < 0.05). In contrast, a comparison of the Control and SCFPa treatments revealed that the relative abundances of 70 bacterial taxa were higher in one of the treatments (Fig. 6). The relative abundances of members of Lachnospiraceae, Succinivibrionaceae, Prevotellaceae*, Sharpea, Selenomonas, Megasphaera, Anaerovibrio, Pyramidobacter,* and *Olsenella* were higher in SCFPa treatment compared to the Control treatment during the SARA challenges (*P* < 0.05).

### Predicted functionality of rumen liquid microbiota

**Fig. 7:**
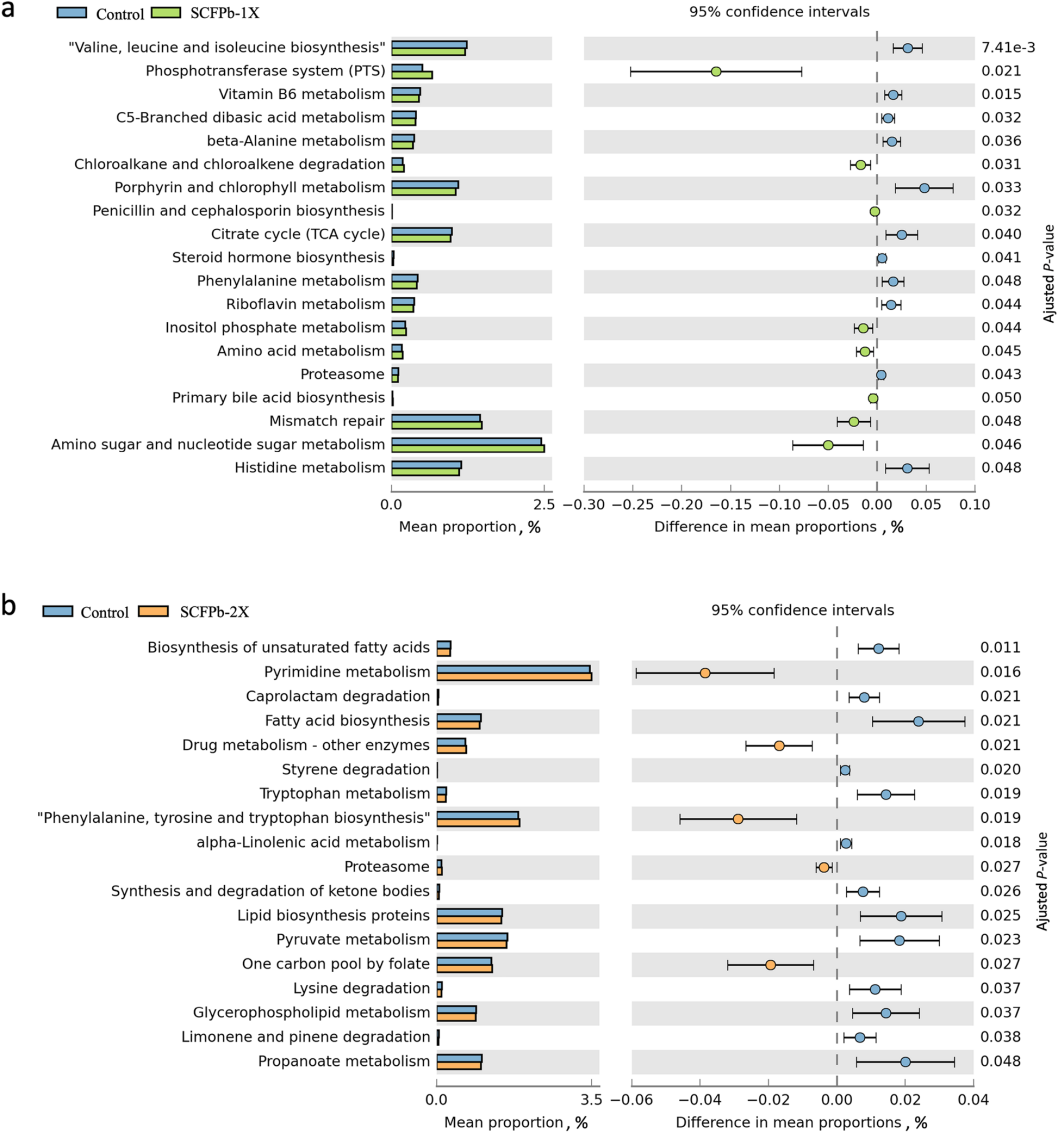
Differences in predicted functions of rumen liquid microbiota between Control and SCFPb-1X and 2X groups. **a** Control vs. SCFPb-1X; **b** Control vs. SCFPb-2X. Functionalities of rumen liquid microbiota were predicted using CowPi. Output was analyzed using STAMP following log_transformation and false discovery rate correction. Significant differences were considered as *P* < 0.05

The predicted functionality of microbiota in rumen liquid are shown in Fig. 7 and Additional files 5–8. Comparisons of these features in rumen liquid associated microbiota between Control and SCFP treatments during both non-SARA and SARA stages showed that the SCFPb-2X treatment reduced 2 predicted carbohydrate metabolic pathways including “propanoate metabolism” and “pyruvate metabolism”, increased 1 predicted amino acid pathway “phenylalanine, tyrosine and tryptophan biosynthesis”, and inhibited 6 predicted lipid pathways including “biosynthesis of unsaturated fatty acids”, “fatty acid biosynthesis”, “glycerophospholipid metabolism”, “lipid biosynthesis proteins”, “synthesis and degradation of ketone bodies”, and “alpha-linolenic acid metabolism” compared to the Control (*P* < 0.05, Fig. 7b). The SCFPb-1X treatment did not impact the predicted lipid metabolic pathways, but it increased 2 predicted carbohydrate pathways including “amino sugar and nucleotide sugar metabolism”, and “inositol phosphate metabolism” metabolic pathways and 1 predicted amino acid pathway “amino acid metabolism” metabolic pathway, and reduced 2 predicted carbohydrate pathways including “C5-branched dibasic acid metabolism” and “citrate cycle (TCA cycle)” and 3 predicted amino acids metabolic pathways including “valine, leucine and isoleucine biosynthesis”, “histidine metabolism” and “phenylalanine metabolism” compared to the Control (*P* < 0.05, Fig. 7a). No differences were observed between the Control and SCFPa treatment in the predicted functionality of microbiota in rumen liquid digesta.

### Association of SCFP supplementation and SARA challenges with co-occurrence patterns of rumen liquid microbiota

**Fig. 8:**
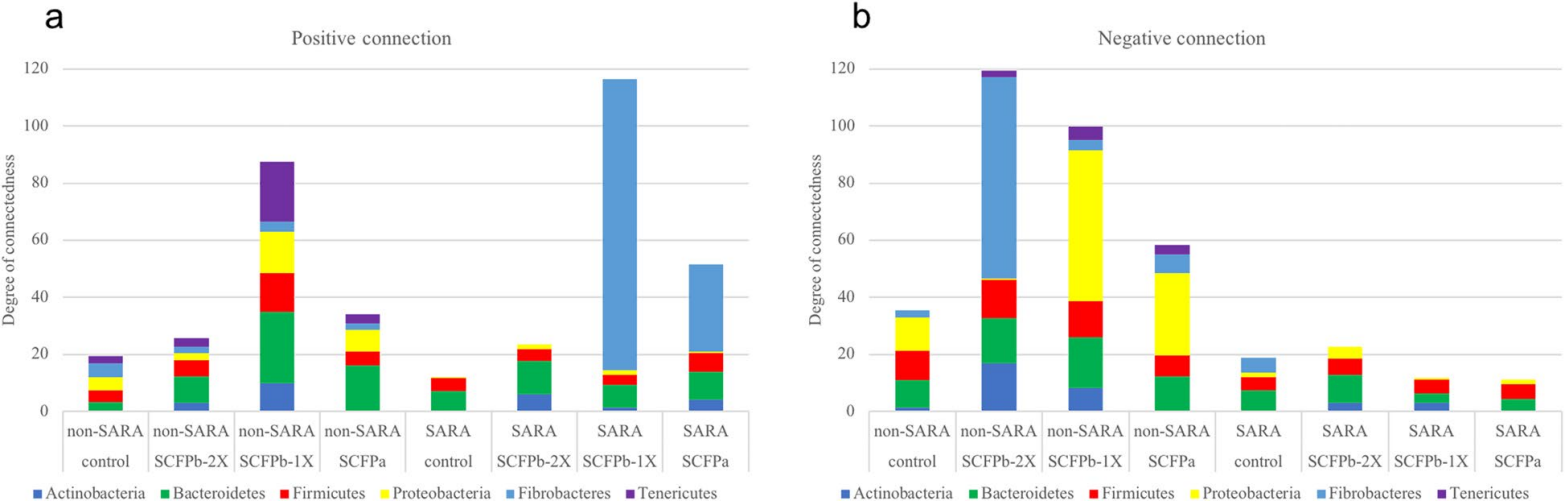
Microbial interaction networks. The degree of connections for each phylum was normalized by dividing the total number of positive and negative edges observed for each phylum by their relative abundance in the community. Normalized **a** positive connections, and **b** negative connections for the dominant bacteria phyla within each treatment group during non-SARA (Pre-SARA1, Post-SARA1 and Post-SARA2) and SARA (SARA1/1, SARA1/2, SARA2/1, SARA2/2) stages

The SARA challenges reduced the total number of significant associations among different microbial taxa. Although the proportions of abundant bacterial phyla did not differ within each SCFP treatment group during SARA stages (Table 1), the relative degrees of connectedness (total number of positive and negative edges observed for each phylum divided by its relative abundance in the community) varied greatly among SCFP treatments (Fig. 8a and b).

Averaged across non-SARA stages, the degrees of positive and negative connections were higher in SCFP supplemented cows, compared to Control cows (Fig. 8a). Of all major phyla, Bacteroidetes had the highest degree of positive connections in SCFP supplemented cows. In descending order, Bacteroidetes, Firmicutes had a high number of positive connections in SCFPb-2X supplemented cows, whereas Tenericutes, Proteobacteria and Firmicutes had a high number of positive connections in SCFPb-1X supplemented cows, and Proteobacteria and Firmicutes had high number of positive connections in cows on the SCFPa treatment (Fig. 8a). In contrast, in Control cows, the number of positive connections was equal for the Actinobacteria, Proteobacteria, Firmicutes and Bacteroidetes phyla. Fibrobacteres contributed to the largest number of negative connections in the SCFPb-2X treatment, whereas in the SCFP-1X and SCFPa treatments, Proteobacteria was the major contributor to negative connections (Fig. 8b). Negative connections in Control cows were contributed equally by the phyla Proteobacteria, Firmicutes and Bacteroidetes.

Averaged across SARA challenges, Bacteroidetes had the highest number of positive connections for cows in the Control and SCFPb-2X treatments, whereas Fibrobacteres had the highest number of positive connections in the SCFPa and SCFPb-1X treatments. The number of positive connections was 2.5 times and 6 times greater in SCFPb-1X and SCFPa treatments, respectively, compared to the Control treatment. All treatment groups showed a smaller number of negative connections among bacterial taxa during the SARA stages compared to the non-SARA stages.

**Fig. 9:**
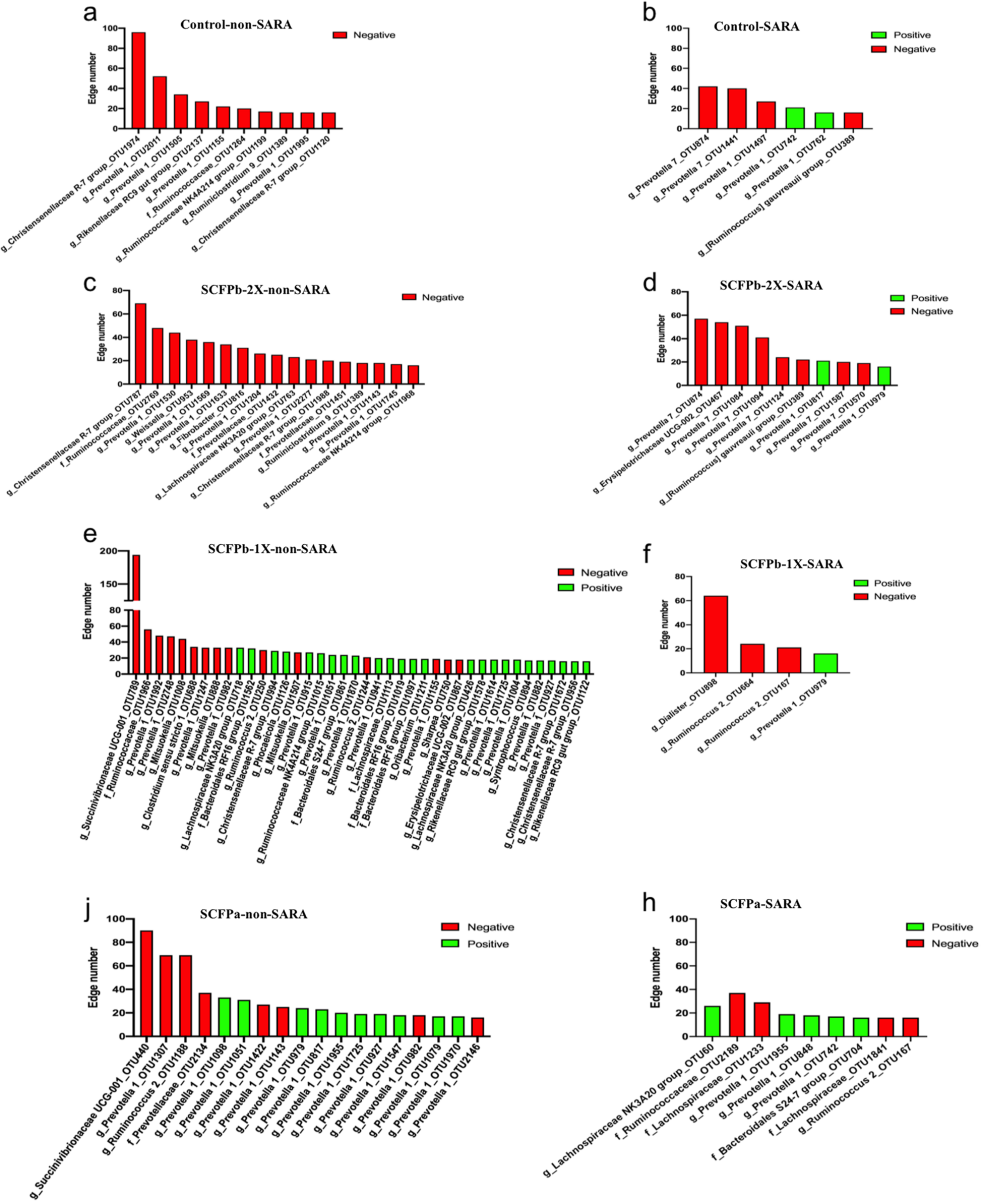
Distribution of hub taxa within each treatment group during non-SARA and SARA stages. Hub taxa were identified as ASVs with more than 15 connections with other members of rumen liquid microbiota within each treatment group during non-SARA (Pre-SARA1, Post-SARA1 and Post-SARA2) and SARA (SARA1/1, SARA1/2, SARA2/1, SARA2/2) stages. **a** and **b** control; **c** and **d** SCFPb-2X; **e** and **f** SCFPb-1X; **j** and **h** SCFPa. Red color represents negative connections while green represents positive connections with other community members

Hub taxa were identified as taxa with the highest number of positive or negative connections with other members of the community (> 15 connections; Fig. 9). The SARA challenges reduced the number of hub taxa in and rumen fluid of all treatments. During non-SARA stages, Control cows had several negatively connected hub taxa from Bacteroidetes (4 taxa belonging to genus *Prevotella* and 1 taxon from Rikenellaceae RC9 family) and from Firmicutes (2 taxa of Christensenellaceae R-7 family, 2 taxa of Ruminococcaceae family and 1 taxon of genus *Ruminiclostridium*) (Fig. 9a). The SCFPb-2X treatment had negatively connected hub taxa from the phyla Bacteroidetes (7 taxa belonging to *Prevotella* genus or Prevotellaceae family), Firmicutes (2 taxa of Ruminococcaceae family*,* 2 taxa of Christensenellaceae R-7 family*,* 1 taxon of Lachnospiraceae NK3A20 family, and 1 taxon of each of *Weissella* and *Ruminiclostridium* genera) and Fibrobacteres (1 taxon from *Fibrobacter* genus) (Fig. 9c). In contrast, the SCFPb-1X treatment resulted in a combination of negatively and positively connected hub taxa from the phyla Proteobacteria (1 negatively connected taxon of Succinivibrionaceae UCG-001 family), Bacteroidetes (9 taxa including 5 negatively connections from the *Prevotella* genus, 4 positively connections of Bacteroidales RF16 class, and 2 positive connections from Rikenellaceae RC9 family), and Firmicutes (1 positively connected taxon of Ruminococcaceae NK4A214 family, 3 positively connected taxa of Christensenellaceae R-7 family, 2 positively connected taxa of Lachnospiraceae NK3A20 family, 2 negative connected taxa of *Mitsuokella* genus, and 1 negative connected taxon from genus *Sharpea*) (Fig. 9e). Similar to the SCFPb-1X treatment, the SCFPa treatment resulted in a combination of negatively and positively connected hub taxa. However, the number of these hub taxa was less than that of the SCFPb-1X treatment (18 vs. 40). The hub taxa in the SCFPa treatment were from the phyla Proteobacteria (1 negatively connected taxon of Succinivibrionaceae UCG-001 family), Bacteroidetes (5 negatively and 10 positively connected taxa from genus *Prevotella*), and Firmicutes (1 negative connected taxon belonging to *Ruminococcus* genus) (Fig. 9g).

During the SARA challenges, hub taxa in all treatments were members of the Bacteroidetes and Firmicutes phyla. The Control treatment was characterized by 3 negatively connected and 2 positively connected taxa belonging to genus *Prevotella* and 1 negatively connected taxon of *Ruminococcus gauvreauii* (Fig. 9b). The SCFPb-2X treatment was characterized by 7 negatively taxa and 1 positively connected taxon from genus *Prevotella,* and 2 negatively connected taxa, including one from *Ruminococcus gauvreauii* and one from the Erysipelotrichaceae UCG-002 family (Fig. 9d). The SCFPb-1X treatment resulted in 2 negatively connected taxa from the *Ruminococcus* genus, 1 negatively connected taxon of the *Dialister* genus, and one positively connected taxon from genus *Prevotella* (Fig. 9f)*.* Finally, the SCFPa treatment resulted in 1 positively and 2 negatively connected taxa from the Lachnospiraceae family*,* 2 negatively connected taxa from the Ruminococcaceae family*,* and 3 positively connected taxa belonging to genus *Prevotella* (Fig. 9h)*.*

### Correlation between hub taxa and diversity metrics of rumen liquid microbiota as well as with rumen fermentation characteristics

**Fig. 10:**
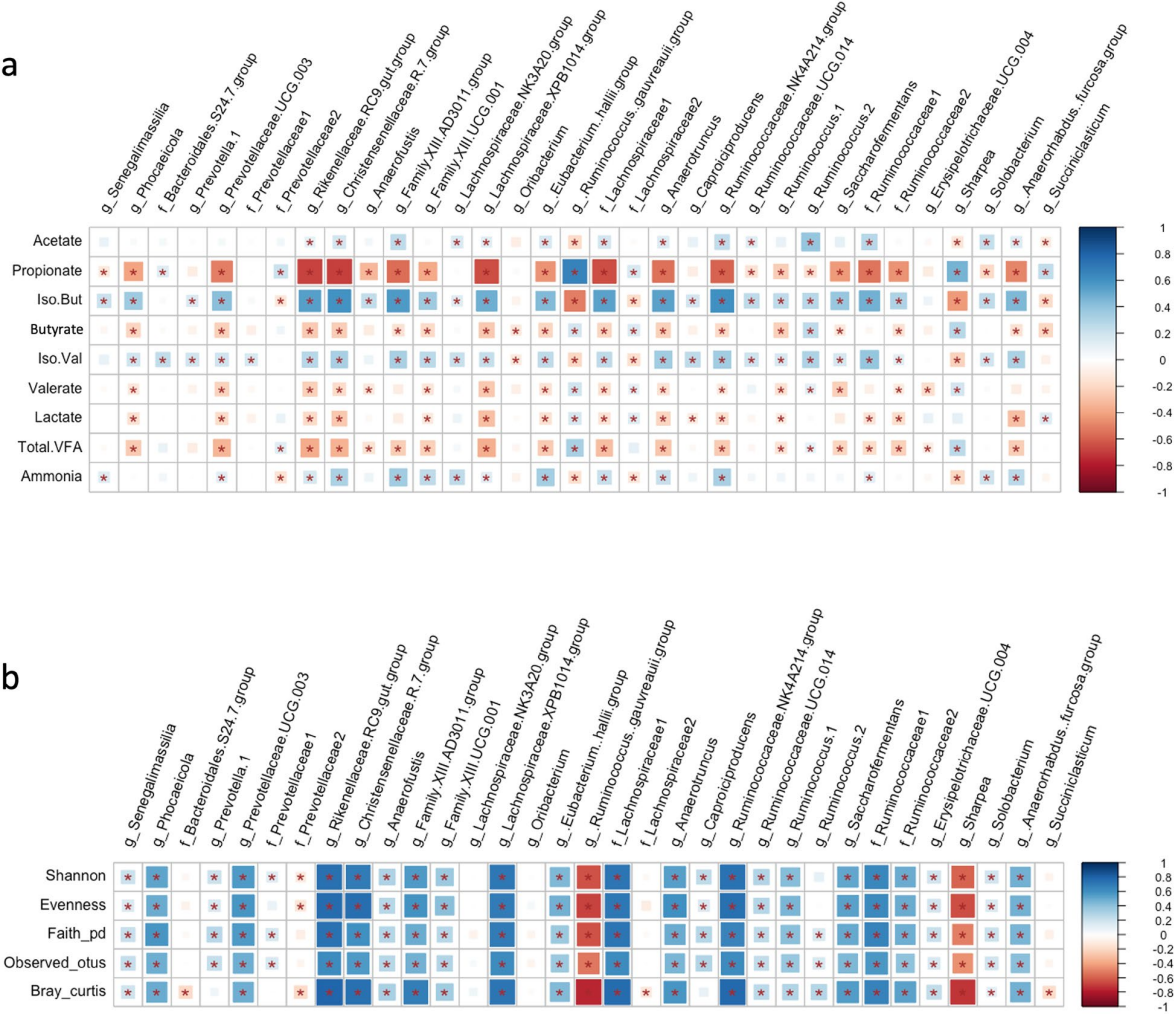
Correlation between hub taxa and microbial diversity metrices and rumen fermentation characteristics. Spearman’s correlation coefficient was used to explore the relationships between the relative abundances of rumen liquid hub taxa and community α-diversity (Shannon, Evenness, Faith_PD and Observed_Features), β-diversity (Bray-Curtis dissimilarities and unweighted UniFrac distances) and rumen fermentation characteristics (VFA and ammonia concentrations). ^*^P < 0.05, ^**^P < 0.01. The color ramp and the size of the squares indicate the type and strength of the Spearman’s correlation coefficient (rho): rho =1 showing strong positive correlation and rho = −1 showing strong negative correlation between the two parameters

In order to evaluate the influence of hub taxa, the relationships between hub taxa, diversity metrics of rumen liquid microbiota and rumen VFAs and ammonia concentrations [31] were determined. A group of taxa from Bacteroidetes, including the Bacteroidales RF16 family and the Rikenellaceae RC9 family*,* taxa from Firmicutes, including the Christensenellaceae R-7 family*,* taxa from the Ruminococcaceae NK4A214 family and unclassified Ruminococcaceae family*,* as well as one taxon from genera *Fibrobacter* and *Prevotella* 1 were negatively correlated with the concentrations of propionate, butyrate, and lactate in rumen liquid digesta (Fig. 10a and b). However, these taxa were positively correlated with the acetate and ammonia concentrations in rumen fluid and the α-, β-diversity metrics of rumen liquid microbiota. Another group of taxa from Bacteroidetes, including members of the *Prevotella* 7 and *Ruminococcus gauvreauii* genera, from Firmicutes, including members of *Sharpea, Dialister* and *Mitsuokella* genera, and from Proteobacteria, including the Succinivibrionaceae UCG-001 family were positively correlated with propionate concentrations in rumen fluid, but negatively correlated with acetate and ammonia concentrations in rumen fluid and the α-, β-diversity metrics of rumen liquid microbiota.

## Discussion

The diversity, richness and taxonomic composition of microbial communities affect their functional properties, such as robustness and resilience [45]. Also, the relationships among microbiota are important to the diversity and can affect the stability of microbial communities. Here, we determined the effects of grain-based SARA challenges and SCFP supplementation on diversity, composition, co-occurrence patterns and predicted functional potential of the microbiota in rumen liquid digesta.

### Effects of grain-based SARA challenges on diversity, composition and predicted functionality of rumen liquid microbiota

In our companion study [31], we showed that the SARA challenges in this trial resulted in a rumen pH depression between 5.2 and 5.6 for more than 180 min/d, which is a commonly accepted threshold of SARA [22, 46]. The SARA challenges included an increase in the dietary starch content from 17.6% to 27.9% DM and reduced the dietary NDF content from 55.4% to 48.1 % DM [31, 32]. As a result, we expected that the relative abundances of fibrolytic and pH sensitive bacteria would decrease, while those of amylolytic and pH tolerant bacteria to increase in the rumen liquid microbiome during SARA [13, 14, 29]. Gram-negative bacteria shed the endotoxin LPS during rumen pH depressions and can trigger an inflammatory response [22, 46]. In a second companion manuscript [32], we reported that SARA was induced successively and that is triggered inflammatory responses.

Rumen microbial members are essential for ruminants’ feed utilization, including digestion and fermentation of fiber, and conversion of feed into absorbable compounds [47]. The reduction in richness and diversity of the microbiome of rumen liquid digesta during the SARA challenges agrees with previous studies [7, 13, 48], suggested that the robustness of rumen bacterial community was reduced by these challenges. Differently from previous studies, we used 32 animals in this study in a complete randomized design, which avoided the carry-over effects might happened in Latin square or crossover experimental designs in other studies [7, 48, 49]. Meanwhile, we observed the temporal dynamics of the microbiome of rumen liquid digesta for 16 weeks, which was much longer than that in previous studies [13, 49, 50]. Our data indicated that SARA can reduce diversity frequently. Analysis of PERMANOVA and PERMDISP of Bray-Curtis distances of rumen liquid microbial communities also demonstrated that SARA challenges affected the taxonomic composition and beta diversity of the rumen liquid microbiota. As in previous studies, we found that Firmicutes, Bacteroidetes and Proteobacteria were the dominant phyla of rumen liquid microbiota [29, 51]. In contrast to earlier studies [13, 29, 49, 52], we observed the SARA challenges performed at 5 and 8 weeks after parturition tended to reduce the relative abundance of Firmicutes while not affecting that of Bacteroidetes. In mammalian species, several taxa of the Bacteroidetes are considered to be primary degraders of complex polysaccharides, as they are more efficient in fermenting these compounds compared with members of Firmicutes [53, 54]. It has also been reported that Bacteroidetes are more abundant in grain-fed cows and that their abundance may decrease during SARA challenges [29, 49, 52]. In our study, several taxa that were recognized as *Prevotella*, which is the dominant genus of family Prevotellaceae in Bacteroidetes, responded differently to SARA. One of the most abundant taxa within this genus, *Prevotella 7,* was positively correlated to the concentration of propionate in rumen digesta, and negatively related to the richness and evenness of the rumen liquid microbial community. Similarly, Zhang et al. [50] found the relative abundance of this genus is positively related to concentration of total VFAs while negatively related to rumen pH. We found the relative abundance of this genus increased during both SARA challenges, indicating its positive correlation with SARA. Besides, hub taxa in both non-SARA and SARA stages of our study mostly belonged to *Prevotella*, which has both positive and negative connections with other members of the rumen liquid microbiota. Therefore, *Prevotella* plays a central role in Bacteroidetes function during SARA. As reported, Prevotellaceae is one of the most abundant family in Bacteroidetes, some members of which can utilize several substrates such as starch, protein and peptides, and generate a wide range of end products, including acetate, succinate, and propionate [55, 56]. The genus *Prevotella* within this family carries a wide range of functional capacities including cellulolytic, amylolytic and fibrolytic activities [29, 57, 58]. The SCFP treatment or SARA did not affect the Firmicutes:Bacteroidetes ratio. This together with the lack of changes in relative abundance of Bacteroidetes suggested that feed efficiency may not be affected [52, 59]. Meanwhile, we found that the abundance of Proteobacteria phylum increased during the SARA challenges, which contrasted with the findings of Tun et al. [29]. Khafipour et al. [7] found that the relative abundance of Firmicutes in the rumen liquid digesta was increased in cows that responded severely to a grain-based SARA challenge, but not in cows that responded moderately to this challenge. This discrepancy among studies, may be due to the difference in dietary starch percentage, stage of lactation, experimental design, sampling time and methods, sequence primers and the methodologies used in 16S rRNA sequencing [29, 49].

Rumen pH depressions during SARA challenges affected the growth of bacterial species that are sensitive to acidic environment. The growth of several fibrolytic bacteria, such as members of Christensenellaceae R-7 group and Lachnospiraceae that are strictly anaerobes [60] were suppressed in our study. The family Ruminococcaceae contains some of the major fibrolytic bacteria within the Firmicutes phylum. The relative abundances of members from this family including unclassified Ruminococcaceae and *Ruminococcus* were decreased by SARA [50]. In partial agreement, we observed that grain-based SARA reduced the abundances of the fibrolytic members including Ruminococcaceae NK4A214 group, *Papillibacter*, Christensenellaceae R-7 group and unclassified Lachnospiraceae. Several members of Succinivibrionaceae can produce succinate, which is the precursor of the propionate that is a substrate for gluconeogenesis [56]. Propionate is the major source of glucose in ruminants [61, 62]. However, too much propionate causes the decrease of milk fat synthesis [63–65]. We observed that the relative abundance of Succinivibrionaceae UCG-001 was positively correlated with the rumen concentrations of propionate, lactate and total VFAs, and negatively correlated with the richness and evenness of the rumen microbial community. In our study, the SARA challenges increased the rumen concentration of propionate [31], as well as the relative abundance of Succinivibrionaceae UCG-001. This may explain some of the adverse effects of SARA on richness and diversity of rumen microbiota and milk fat production*.*

Several amylolytic microorganisms, including the lactate producer *Streptococcus bovis*, are tolerant to the rumen pH depressions caused by SARA [49, 66]. The reason that we did not find an effect of SARA on the relative abundance of this bacterium may be due to its low relative abundance and the SARA conducted in our study was not as severe as other studies.

Several members of the family Lachnospiraceae are producers of butyrate [67]. The production of butyrate provides energy for some microbes and host epithelial cells [68]. It has been reported that the concentrations of butyrate in the rumen are positively related to feed efficiency, which may be due to the increase in the supply of energy to the host animal [5]. However, other studies reported that a higher abundance of this family may increase butyrate metabolism and reduce feed efficiency [56, 69]. In the current study, we detected that the relative abundance of unclassified Lachnospiraceae was positively correlated with the butyrate concentration in the rumen. This may suggest the contribution of this family to ruminal butyrate production. Due to large number of genera within this family that produce butyrate, and the limitation of 16S rRNA sequencing in identifying taxa at the species level, the relationships between this family and feed efficiency needs further research.

Previous studies have shown that inefficient dairy cows have higher nitrogen metabolism activities compared to efficient cows [56]. Similarly, from the functional prediction of rumen liquid microbiota, we found that predicted “nitrogen metabolism” was inhibited by SARA, including “phenylalanine, tyrosine and tryptophan biosynthesis”, “alanine, aspartate and glutamate metabolism”, “lysine biosynthesis”, “phenylalanine metabolism”, “glycine, serine and threonine metabolism” and “arginine and proline metabolism”, suggesting that SARA might reduce the feed efficiency. However, more research is required to confirm this due to the limitation and inaccuracy of 16S rRNA sequencing. We observed that rumen ammonia-nitrogen was reduced by the SARA challenges [31]. This may indicate that the proteolytic bacteria were suppressed during SARA as starch content increased, and the ammonia from protein degradation by these proteolytic bacteria, which can be utilized by fibrolytic bacteria were also decreased [70–72]. This suggested that the availability of substrates for microbial protein synthesis is reduced during SARA challenges.

### Effects of SCFP supplementation on diversity, composition and predicted functionality of rumen liquid microbiota

Several SCFP have been used to improve the rumen function and feed utilization, as they are rich in several constituents such as vitamins, short-chain fatty acids (SCFAs), microbial cell fractions, functional proteins, extracellular polysaccharides, cell lysates, antioxidants and β-glucans that promote the growth of lactate-utilizing bacteria, cellulolytic bacteria and fungi in the rumen, hence, stabilizing the ruminal pH [27, 29, 30, 73, 74]. Studies have reported that supplementation with SCFP helped to maintain rumen pH during high-starch diet feeding and under grain-based SARA challenges [75–77]. Our companion papers showed that SCFP supplementation, and especially SCFPb-2X, attenuated the decrease of rumen pH, and NDF digestibility, and decreased the rumen concentration of free LPS and the acute phase response, caused by SARA challenges [31, 32]. This indicated that SCFP attenuated several adverse effects of SARA.

Previous studies suggested that SCFP can improve milk production and feed efficiency and stabilize rumen fermentation by enhancing the growth of fiber-digesting and lactic acid-utilizing bacteria [30, 74, 78, 79]. In addition, Tun et al. [29] demonstrated that SCFP (XPC) attenuate the reduction of richness and diversity and changes of the β-diversity of rumen microbiota during SARA challenges. Rumen microbial members require specific substrates and vary in functionality, hence, a more diverse community may imply more efficient use of feed, as the functionality of taxa that are reduced by nutritional challenges may be replaced by taxa with similar functionality that are less affected by these challenges [13, 80]. In agreement, the current study showed that cows on the SCFPb-2X treatment tended to have higher evenness of the rumen microbiota than cows on the Control treatment during the first SARA challenge (SARA1). This showed the dose benefit of SCFPb, in that only in 2X supplementation can be sufficient to reduce the microbial variation caused by SARA challenges. Additionally, our results showed that the changes of β-diversity of rumen liquid microbiota were less in SCFP treatment groups than in the Control treatment group, which indicated that SCFP supplementation supports the robustness and stability of the microbial community. Our study also found that during SARA challenges, SCFPb-2X stimulated the growth of several fibrolytic bacteria, including Ruminococcaceae NK4A214 group, *Papillibacter*, Christensenellaceae R-7 group and unclassified Lachnospiraceae, and the lactate utilizer *Selenomonas* [81] compared with the Control treatment. In contrast, the relative abundances of Ruminococcaceae NK4A214 group, Christensenellaceae R-7 group and unclassified Lachnospiraceae were negatively correlated with ruminal concentrations of propionate and butyrate that were increased during SARA, while being positively correlated with the richness and evenness of the microbial community. Although SCFPb-2X supplementation contributed to maintaining the diversity of the rumen microbiota during SARA challenges, this did not affect the rumen concentrations of propionate and butyrate. In addition, the SCFPa treatment increased the relative abundances of *Selenomonas* and *Megasphaera,* which are main lactate utilizers [81]. The latter demonstrated its effect on preventing the accumulation of lactic acid and severe rumen acidosis.

We monitored the longitudinal shifts in the rumen liquid bacteria over the 16 experimental weeks, and found that supplementation with SCFP attenuated the fluctuations in the relative abundances of Bacteroidetes and Firmicutes phyla that resulted from the SARA challenges. For Proteobacteria and Tenericutes, only SCFPb-2X attenuated this fluctuation. Although the relative abundances of the Proteobacteria and Tenericutes phyla were lower than those of the Bacteroidetes and Firmicutes phyla, Proteobacteria and Tenericutes showed strong connections with other taxa. Thus, SCFPb-2X stabilized the populations of both foundation and keystone members of rumen liquid microbiota during grain-based SARA challenge. In addition to the higher evenness during SARA1, SCFPb-2X also improved the robustness and stability of the rumen liquid microbial community during SARA challenges in our study. A more diverse community with complex biotic interactions among commensal microbes has a stronger stability and capability to withstand exogenous perturbations. Such community has higher and more diverse activities, can utilize feed compounds and generate more diverse metabolic products compared to a less diverse community [56, 82–84]. We observed that SCFPb-1X and SCFPa supplementation promoted the positive connectedness in the microbial network in liquid rumen digesta during both SARA and non-SARA experimental stages. Both SCFPb-2X and SCFPa supplementation increased the diversity of hub taxa during both non-SARA and SARA stages, while SCFPb-1X only increased the diversity of hub taxa during non-SARA stages suggesting that SCFPb-1X has a smaller effect on the composition of the rumen liquid microbiota. Supplementation with SCFPb-2X promoted populations of taxa (*Prevotella* and *unclassified Bacteroidales RF16*) that showed strong associations with diversity metrics which mostly belonged to Bacteroidetes, whereas SCFPb-1X and SCFPa promoted taxa that mostly belonged to Firmicutes during SARA challenges. In contrast, SCFPb-2X was more beneficial in increasing rumen fermentation when high-grain diets were supplied.

An increase in the intake of starch increases the production and absorption of propionate [85]. It has been reported that SCFP (XP yeast culture) reduced the ruminal digestibility and fermentation of starch when cows were fed large amounts of fermentable starch [75]. In agreement, we observed SCFPb-2X increased the proportion of Succinivibrionaceae UCG-001 during SARA challenges, which agrees with the observation from previous study [25]. Meanwhile, we also observed that the SCFPb-2X treatment had a lower predicted “propanoate metabolism” compared with the control treatment during SARA, which demonstrated that the effect of SCFPb with higher dose on reducing starch digestibility. Furthermore, SCFPb-1X reduced several nitrogen metabolism pathways, including “valine, leucine and isoleucine degradation”, “beta-alanine metabolism”, “phenylalanine metabolism” and “histidine metabolism”. Supplementation with SCFPb-2X, however, reduced “tryptophan metabolism” and “lysine degradation”. Valine, leucine and isoleucine are important in microbial protein synthesis, and microbial protein provides most precursors for the synthesis of milk protein in the mammary gland [58, 86]. Xue et al. [58] reported that “valine, leucine and isoleucine degradation”, “lysine degradation” and “phenylalanine metabolism” were enriched in the microbiome of cows with low milk protein yields. Therefore, SCFPb supplementation may help to decrease the degradation of the microbial protein and promotes more microbial protein supply to the small intestine during grain-based SARA challenges.

## Conclusions

In this study, we monitored the longitudinal microbial changes in rumen liquid for a long period with a large number of cannulated dairy cows, which was not common in previous studies. Two subsequent grain-based SARA reduced the richness and diversity and changed the β-diversity of the microbial community in rumen liquid with no obvious differences between two SARA challenges. The relative abundances of the predominant phyla Firmicutes, Bacteroidetes, Proteobacteria and Tenericutes in rumen liquid microbiota experienced fluctuations during SARA challenges and during the recovery stages following these challenges. Grain-based SARA challenges also reduced the biotic interaction between microbes, hub taxa diversity and the robustness of the microbiota in rumen liquid digesta. Additionally, grain-based SARA challenges reduced the growth of several fibrolytic bacteria and several amino acids synthesis pathways, which may reduce the microbial protein and milk protein synthesis. Supplementation with SCFPb, and more profoundly SCFPb-2X, attenuated above negative effects of SARA on rumen liquid microbiome.

Furthermore, supplementation with SCFPb-2X reduced predicted “propionate metabolism”, which may better control the starch digestibility when cows consume too much fermentable carbohydrate, hence, preventing further reduction in rumen pH. Our results indicated that grain-based SARA challenges altered the microbial environment in rumen liquid digesta and SCFP attenuated negative effects of SARA challenges with SCFPb-2X having the greatest impact among all SCFP tested. Due to the limitation of the 16S rRNA sequencing to classify taxa at the species level and of the databases for rumen microbiome when using CowPi to predict the microbial functionality, further metagenomic assembly based (MAGs) approaches are needed to investigate the association among microbes, and the gastrointestinal health of the host animal, especially when the animals are exposed to nutritional and physiological challenges.

### Supplementary Information

**Additional file 1**. Alpha-rarefaction curves.

**Additional file 2**. Effects of treatment and stage of SARA induction on alpha-diversity of rumen liquid microbiome.

**Additional file 3**. Longitudinal shifts in Proteobacteria and Tenericutes proportions in rumen liquid microbiota.

**Additional file 4**. Concepts of stability, robustness, and resilience of rumen liquid microbiota.

**Additional file 5**. Differences in predicted microbial metabolic pathways between nonSARA (Pre-SARA1, Post-SARA1 and Post-SARA2) and SARA (SARA1/1, SARA1/2, SARA2/1, SARA2/2) stages in control group.

**Additional file 6**. Differences in predicted microbial metabolic pathways between nonSARA (Pre-SARA1, Post-SARA1 and Post-SARA2) and SARA (SARA1/1, SARA1/2, SARA2/1, SARA2/2) stages in SCFPb-2X group.

**Additional file 7**. Differences in predicted microbial metabolic pathways between nonSARA (Pre-SARA1, Post-SARA1 and Post-SARA2) and SARA (SARA1/1, SARA1/2, SARA2/1, SARA2/2) stages in SCFPb-1X group.

**Additional file 8**. Differences in predicted microbial metabolic pathways between nonSARA (Pre-SARA1, Post-SARA1 and Post-SARA2) and SARA (SARA1/1, SARA1/2, SARA2/1, SARA2/2) stages in SCFPa group.

## Data Availability

The datasets used and/or analyzed during the current study are available from the corresponding author on reasonable request. The rumen liquid sequencing data were deposited into NCBI Sequence Read Archive (SRA) under the Bioproject ID: PRJNA1114003 with submission ID: SUB14463266.

## Abbreviations

ASV: Amplicon sequence variant
CAZyme: Carbohydrate active enzymes
CoNet: Correlation network
CP: Crude protein
CSS: Cumulative sum scaling
DFM: Direct fed microbials
eNDF: Effective neutral detergent fiber
MetaLonDA: Metagenomic longitudinal differential abundance
NDF: Neutral detergent fiber
nMDS: Non-metric multidimensional scaling
PERMANOVA: Permutational multivariate analysis of variance
PERMDISP: Permutational multivariate analysis of dispersion
PICRUSt: Phylogenetic investigation of communities by reconstruction of unobserved states
SAA: Serum amyloid A
SARA: Subacute ruminal acidosis
SCFA: Short-chain fatty acid
SCFP: *Saccharomyces* cerevisiae fermentation product
TMR: Total mixed ration
VFA: Volatile fatty acid
