# Supplementary material for: Postbiotics from Saccharomyces cerevisiae fermentation stabilize microbiota in rumen liquid digesta during grain-based subacute ruminal acidosis (SARA) in lactating dairy cows
Source: J Anim Sci Biotechnol. 2024 Aug 1;15:101. doi: 10.1186/s40104-024-01056-x (PMC11293205; doi:10.1186/s40104-024-01056-x)

**Supplementary information**

**Postbiotics from *Saccharomyces cerevisiae* fermentation stabilize microbiota in rumen liquid digesta during grain-based subacute ruminal acidosis (SARA) in lactating dairy cows**

**Additional file 1** Alpha-rarefaction curves


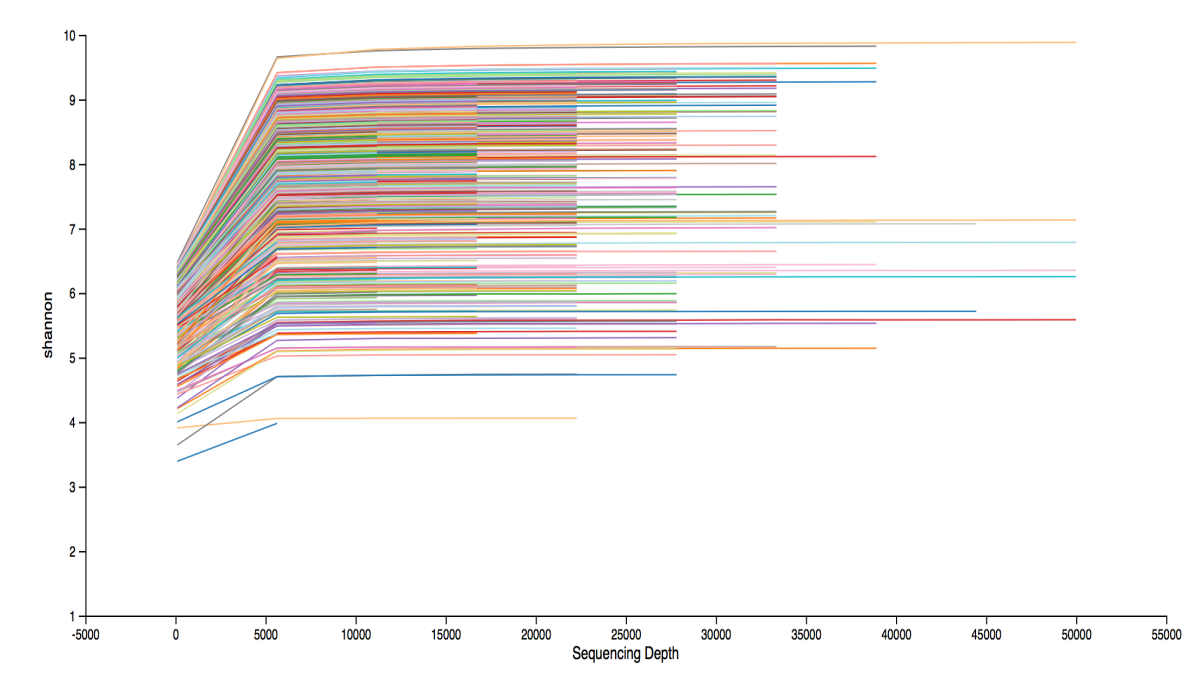


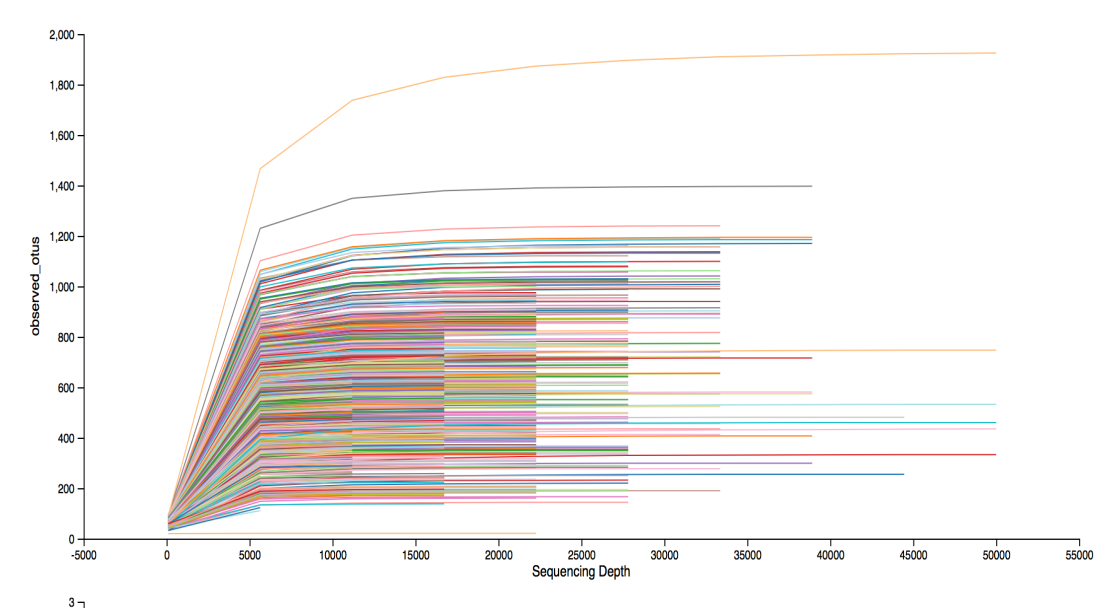

Supplement: Supplementary file 1 — Additional file 1. Alpha-rarefaction curves. [file 40104_2024_1056_MOESM1_ESM.docx]
