# Supplementary material for: Postbiotics from Saccharomyces cerevisiae fermentation stabilize microbiota in rumen liquid digesta during grain-based subacute ruminal acidosis (SARA) in lactating dairy cows
Source: J Anim Sci Biotechnol. 2024 Aug 1;15:101. doi: 10.1186/s40104-024-01056-x (PMC11293205; doi:10.1186/s40104-024-01056-x)
Supplement: Supplementary file 2 — Additional file 2. Effects of treatment and stage of SARA induction on alpha-diversity of rumen liquid microbiome. [file 40104_2024_1056_MOESM2_ESM.docx]

**Supplementary information**

**Postbiotics from *Saccharomyces cerevisiae* fermentation stabilize microbiota in rumen liquid digesta during grain-based subacute ruminal acidosis (SARA) in lactating dairy cows**

| **Additional file 2** Effects of treatment and stage of SARA induction on alpha-diversity of rumen liquid microbiome | | | | | | | | | | | | | | | | | |
| --- | --- | --- | --- | --- | --- | --- | --- | --- | --- | --- | --- | --- | --- | --- | --- | --- | --- |
| **Item** | **Treatment^1^** | **Stage^2^** | | | | | | | | | | | | **SEM** | ***P*-value^3^** | | |
|  |  | **week −4** | **week −1** | **week 1** | **week 3** | **week 4**  **Pre-SARA1** | **week 5**  **SARA1/1** | **week 5**  **SARA1/2** | **week 7**  **Post-SARA1** | **week 8**  **SARA2/1** | **week 8**  **SARA2/2** | **week 10**  **Post-SARA2** | **week 12**  **Post-SARA2** |  | **Treat** | **Stage** | **Int^4^** |
| Shannon’s diversity | Control | 8.78^a^ | 8.77^a^ | 8.54^a^ | 8.64^a^ | 8.53^a^ | 7.33^b^ | 7.05^b^ | 8.52^a^ | 6.95^b^ | 6.41^b^ | 8.56^a^ | 8.52^a^ | 0.11 | 0.62 | <0.0001 | 0.03 |
|  | SCFPa | 8.58^a^ | 8.71^a^ | 8.08^bc^ | 8.80^a^ | 8.76^a^ | 6.52^d^ | 6.68^d^ | 8.60^a^ | 6.32^d^ | 6.73^d^ | 8.53^ab^ | 7.92^c^ | 0.11 |  |  |  |
|  | SCFPb-1X | 8.71^ab^ | 8.66^ab^ | 8.82^a^ | 8.08^bc^ | 8.60^ab^ | 6.55^d^ | 7.17^cd^ | 7.77^c^ | 6.50^d^ | 6.57^d^ | 8.04^c^ | 8.65^ab^ | 0.12 |  |  |  |
|  | SCFPb-2X | 8.49^a^ | 8.50^a^ | 8.29^ab^ | 8.55^a^ | 8.43^a^ | 7.71^bc^ | 7.26^c^ | 8.43^a^ | 7.27^c^ | 7.21^c^ | 8.68^a^ | 8.65^a^ | 0.12 |  |  |  |
|  |  |  |  |  |  |  |  |  |  |  |  |  |  |  |  |  |  |
| Pielou’s Evenness | Control | 0.92^a^ | 0.92^a^ | 0.90^a^ | 0.89^a^ | 0.90^a^ | 0.82^bY^ | 0.81^b^ | 0.91^ax^ | 0.80^b^ | 0.77^b^ | 0.91^ax^ | 0.91^aX^ | 0.01 | 0.13 | <0.0001 | 0.01 |
|  | SCFPa | 0.90^a^ | 0.92^a^ | 0.88^a^ | 0.93^a^ | 0.92^a^ | 0.77^by^ | 0.80^b^ | 0.91^ax^ | 0.75^by^ | 0.80^b^ | 0.91^ax^ | 0.86^ayY^ | 0.01 |  |  |  |
|  | SCFPb-1X | 0.91^a^ | 0.91^a^ | 0.91^a^ | 0.86^bc^ | 0.90^ab^ | 0.78^dy^ | 0.83^cd^ | 0.86^cy^ | 0.79^d^ | 0.80^d^ | 0.87^bcy^ | 0.91^aX^ | 0.01 |  |  |  |
|  | SCFPb-2X | 0.91^a^ | 0.90^ab^ | 0.91^a^ | 0.91^a^ | 0.91^a^ | 0.88^bcxX^ | 0.83^d^ | 0.91^ax^ | 0.83^cdx^ | 0.81^d^ | 0.91^ax^ | 0.92^ax^ | 0.01 |  |  |  |
|  |  |  |  |  |  |  |  |  |  |  |  |  |  |  |  |  |  |
| Faith’s Phylogenetic diversity | Control | 122.97^ab^ | 125.06^ab^ | 116.30^ab^ | 130.62^a^ | 116.76^b^ | 76.74^c^ | 71.70^c^ | 110.09^b^ | 71.94^c^ | 59.83^c^ | 111.43^b^ | 111.75^b^ | 3.92 | 0.54 | <0.0001 | 0.08 |
|  | SCFPa | 117.16^a^ | 117.47^a^ | 99.66^b^ | 121.58^a^ | 116.57^a^ | 64.05^c^ | 62.83^c^ | 109.90^ab^ | 62.82^c^ | 66.75^c^ | 111.65^ab^ | 98.69^b^ | 3.94 |  |  |  |
|  | SCFPb-1X | 120.02^ab^ | 120.06^ab^ | 127.16^a^ | 114.91^ab^ | 119.27^ab^ | 63.53^d^ | 72.29^d^ | 95.70^c^ | 59.28^d^ | 59.50^d^ | 105.34^bc^ | 114.64^ab^ | 4.12 |  |  |  |
|  | SCFPb-2X | 109.99^a^ | 117.54^a^ | 98.69^ac^ | 113.09^a^ | 101.63^a^ | 78.00^b^ | 77.33^b^ | 102.72^a^ | 69.48^b^ | 79.06^bc^ | 117.99^a^ | 111.23^a^ | 4.13 |  |  |  |
|  |  |  |  |  |  |  |  |  |  |  |  |  |  |  |  |  |  |
| Observed features | Control | 757.39^ab^ | 768.50^ab^ | 727.87^ab^ | 832.10^a^ | 732.62^ab^ | 489.62^c^ | 426.76^c^ | 658.37^b^ | 428.00^c^ | 333.13^c^ | 691.37^ab^ | 678.25^b^ | 36.60 | 0.61 | <0.0001 | 0.11 |
|  | SCFPa | 729.87^a^ | 714.01^a^ | 587.87^a^ | 698.21^a^ | 732.12^a^ | 398.83^b^ | 357.00^b^ | 695.62^a^ | 367.12^b^ | 388.50^b^ | 743.25^a^ | 579.50^a^ | 36.83 |  |  |  |
|  | SCFPb-1X | 750.01^a^ | 734.44^a^ | 817.01^a^ | 701.13^ab^ | 740.72^a^ | 382.72^d^ | 448.44^cd^ | 569.29^bc^ | 334.86^d^ | 330.15^d^ | 603.15^abc^ | 728.57^a^ | 38.92 |  |  |  |
|  | SCFPb-2X | 648.44^ab^ | 723.58^a^ | 584.15^bcd^ | 685.04^a^ | 624.86^ab^ | 481.86^cd^ | 486.86^bcd^ | 621.29^abc^ | 442.72^d^ | 481.15^cd^ | 730.72^a^ | 674.56^a^ | 39.04 |  |  |  |
| ^a-d^Means in a row with different superscripts among Stages are different (*P* < 0.05)  ^x,y^Means in a column with different superscripts among treatments are different (*P <* 0.05)  ^X,Y^Means in a column with different superscripts among treatments are tended to be different (0.5 ≤ *P <* 0.1)  ^1^Treatment: Control = 140 g/d ground corn; SCFPa = 14 g/d Diamond V Original XPC mixed with 126 g/d ground corn; SCFPb-1X = 19 g/d NutriTek mixed with 121 g/d ground corn; SCFPb-2X = 38 g/d NutriTek mixed with 102 g/d ground corn.  ^2^Stage: SARA was induced during week 5 (SARA1) and week 8 (SARA2) after parturition. Rumen samples were taken on d 2 (SARA1/1, SARA1/2) and d 5 (SARA2/1, SARA2/2) during each SARA week. Week 4 was considered as Pre-SARA1, week 7 as Post-SARA1, and week 10 and 12 as Post-SARA2  ^3^Statistical analyses were conducted on log_10_-transformed data for Faith’s Phylogenetic diversity, original data for Chao1 and Box-cox data for the rest. Presented means are original values prior to transformation  ^4^Int: Interaction effect of treatment and stage | | | | | | | | | | | | | | | | | |
