# Supplementary material for: Postbiotics from Saccharomyces cerevisiae fermentation stabilize microbiota in rumen liquid digesta during grain-based subacute ruminal acidosis (SARA) in lactating dairy cows
Source: J Anim Sci Biotechnol. 2024 Aug 1;15:101. doi: 10.1186/s40104-024-01056-x (PMC11293205; doi:10.1186/s40104-024-01056-x)
Supplement: Supplementary file 3 — Additional file 3. Longitudinal shifts in Proteobacteria and Tenericutes proportions in rumen liquid microbiota. [file 40104_2024_1056_MOESM3_ESM.docx]

**Supplementary information**

**Postbiotics from *Saccharomyces cerevisiae* fermentation stabilize microbiota in rumen liquid digesta during grain-based subacute ruminal acidosis (SARA) in lactating dairy cows**

**Junfei Guo^1^, Jan C. Plaizier^1,*^, Zhengxiao Zhang^1,†^, Leluo Guan^2^, Ilkyu Yoon^3^, and Ehsan Khafipour^1,‡,*^**

**Additional file 3**. Longitudinal shifts in Proteobacteria and Tenericutes proportions in rumen liquid microbiota. Metagenomic Longitudinal Differential Abundance (MetaLonDA) was used to assess the longitudinal changes in rumen liquid microbial communities as lactation progressed. The OTU table was normalized using cumulative sum scaling (CSS) transformation. The longitudinal profiles in each group were fitted with a negative binomial smoothing spline. The blue color represents control and red color represents SCFPb-2X group. The significant time intervals were identified when *P* < 0.05 after multiple testing corrections using Benjamini-Hochberg False Discover Rate (FDR) estimation. a. Proteobacteria proportion in control vs. SCFPb-2X. b. Tenericutes. proportion in control vs. SCFPb-2X groups. The study was started from 4 weeks before until 12 weeks after parturition. SARA challenges were conducted on wk 5 and wk 8 and rumen samples were taken weekly but twice during SARA weeks (SARA1/1, SARA1/2, SARA2/1, SARA2/2).

**
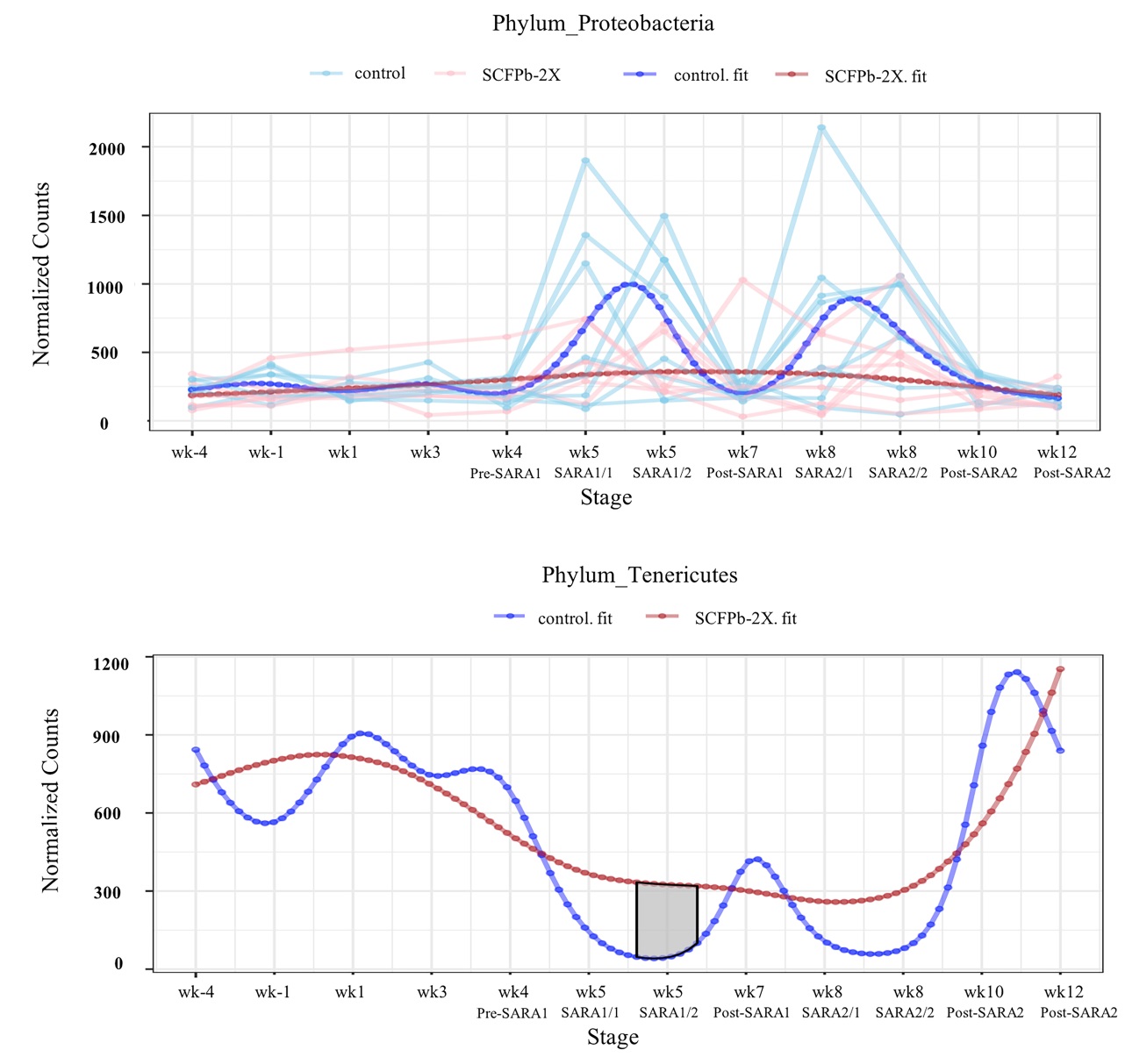
**
