# Supplementary material for: Postbiotics from Saccharomyces cerevisiae fermentation stabilize microbiota in rumen liquid digesta during grain-based subacute ruminal acidosis (SARA) in lactating dairy cows
Source: J Anim Sci Biotechnol. 2024 Aug 1;15:101. doi: 10.1186/s40104-024-01056-x (PMC11293205; doi:10.1186/s40104-024-01056-x)
Supplement: Supplementary file 4 — Additional file 4. Concepts of stability, robustness, and resilience of rumen liquid microbiota. [file 40104_2024_1056_MOESM4_ESM.docx]

**Supplementary information**

**Postbiotics from *Saccharomyces cerevisiae* fermentation stabilize microbiota in rumen liquid digesta during grain-based subacute ruminal acidosis (SARA) in lactating dairy cows**

**Additional file 4** Concepts of stability, robustness, and resilience of rumen liquid microbiota. Longitudinal shifts in the proportion of phylum Firmicutes in Control and SCFPb2X as example for explanation of a. stability, b. robustness, and c. resilience concepts. Stability is defined as day-to-day variation in microbiome. Robustness explains the capacity of microbiome to resist changes that are forced by stressors, e.g., a metabolic challenge such as SARA. Resilience describes the capacity and the time it takes for the microbiome to return to equilibrium (if at all) after it has been pushed away from the equilibrium by the stressors, e.g., recovery after a metabolic challenge such as SARA

**.** **
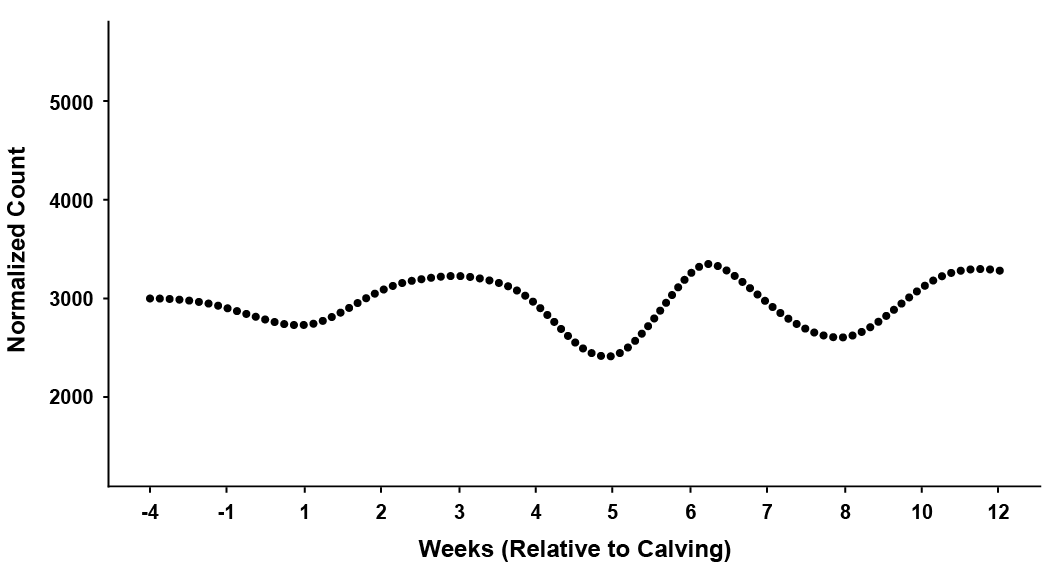

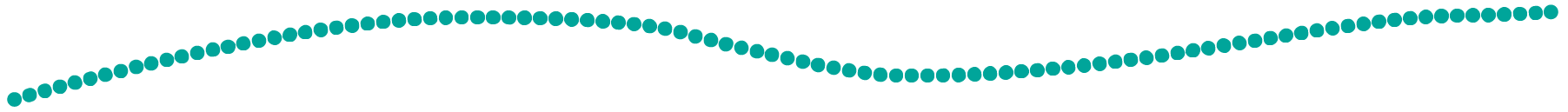
**

Firmicutes proportion

**Weeks post-calving**

Control

**SARA1**

**SARA2**

**1^st^ week**

**post-calving**

SCFPb-2X

-4

0

5

8

12

1

3

2

4

6

7

9

10

**STABILITY**

**
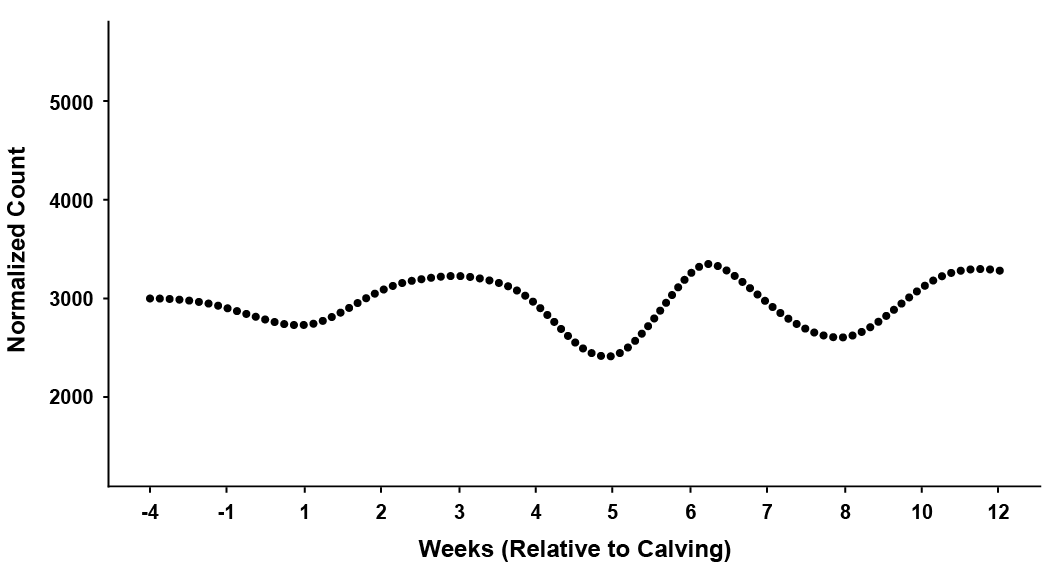

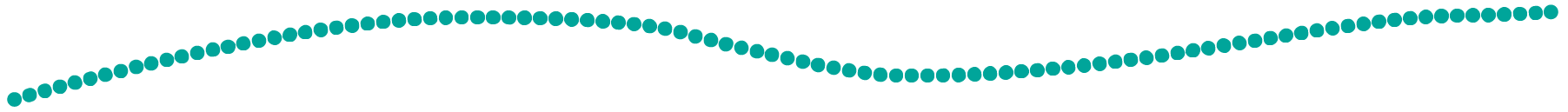
**

Firmicutes proportion

SCFPb-2X

**Weeks post-calving**

Control

**SARA1**

**SARA2**

**1^st^ week**

**post-calving**

-4

0

5

8

12

1

3

2

4

6

7

9

10

**STABILITY**

**ROBUSTNESS**

**ROBUSTNESS**

**ROBUSTNESS**

**
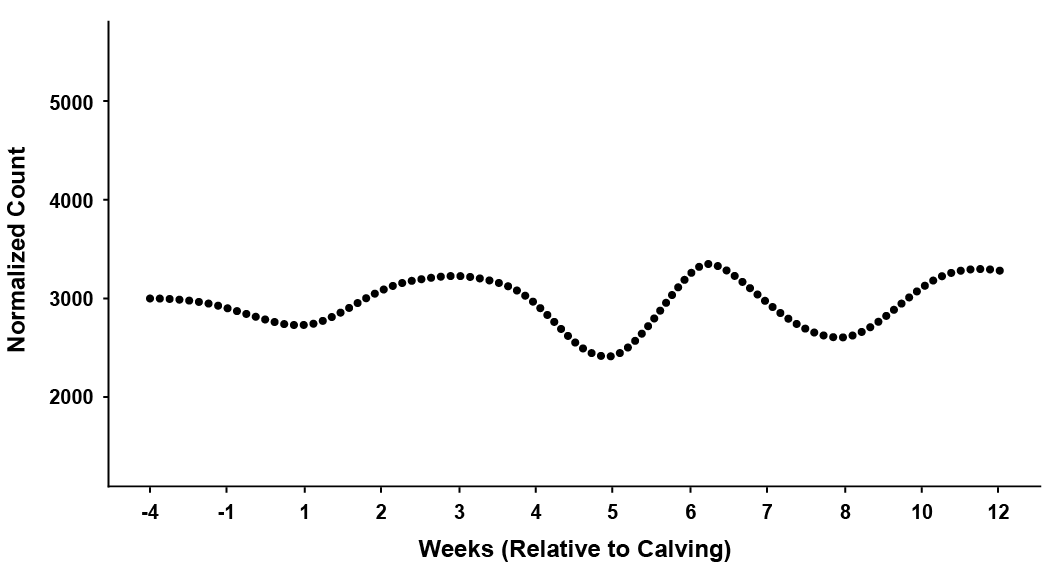

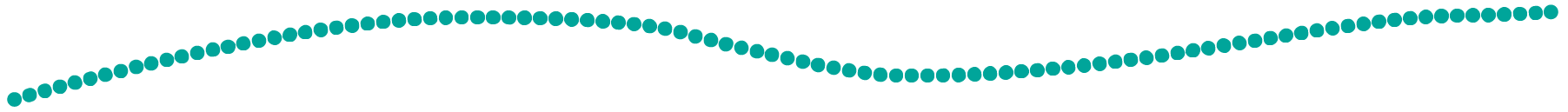
**

Firmicutes proportion

**Weeks post-calving**

Control

**SARA1**

**SARA2**

**1^st^ week**

**post-calving**

-4

0

5

8

12

1

3

2

4

6

7

9

10

**STABILITY**

**RESILIENCE**

SCFPb-2X
