# Supplementary material for: Postbiotics from Saccharomyces cerevisiae fermentation stabilize microbiota in rumen liquid digesta during grain-based subacute ruminal acidosis (SARA) in lactating dairy cows
Source: J Anim Sci Biotechnol. 2024 Aug 1;15:101. doi: 10.1186/s40104-024-01056-x (PMC11293205; doi:10.1186/s40104-024-01056-x)

**Supplementary information**

**Postbiotics from *Saccharomyces cerevisiae* fermentation stabilize microbiota in rumen liquid digesta during grain-based subacute ruminal acidosis (SARA) in lactating dairy cows**

**Additional file 5** Differences in predicted microbial metabolic pathways between nonSARA (Pre-SARA1, Post-SARA1 and Post-SARA2) and SARA (SARA1/1, SARA1/2, SARA2/1, SARA2/2) stages in control group. Functionalities of rumen liquid microbiome were predicted by CowPi and the results were analyzed by STAMP following log transformation and False Discovery Rate (FDR) correction. Significant differences were considered as *P* < 0.05


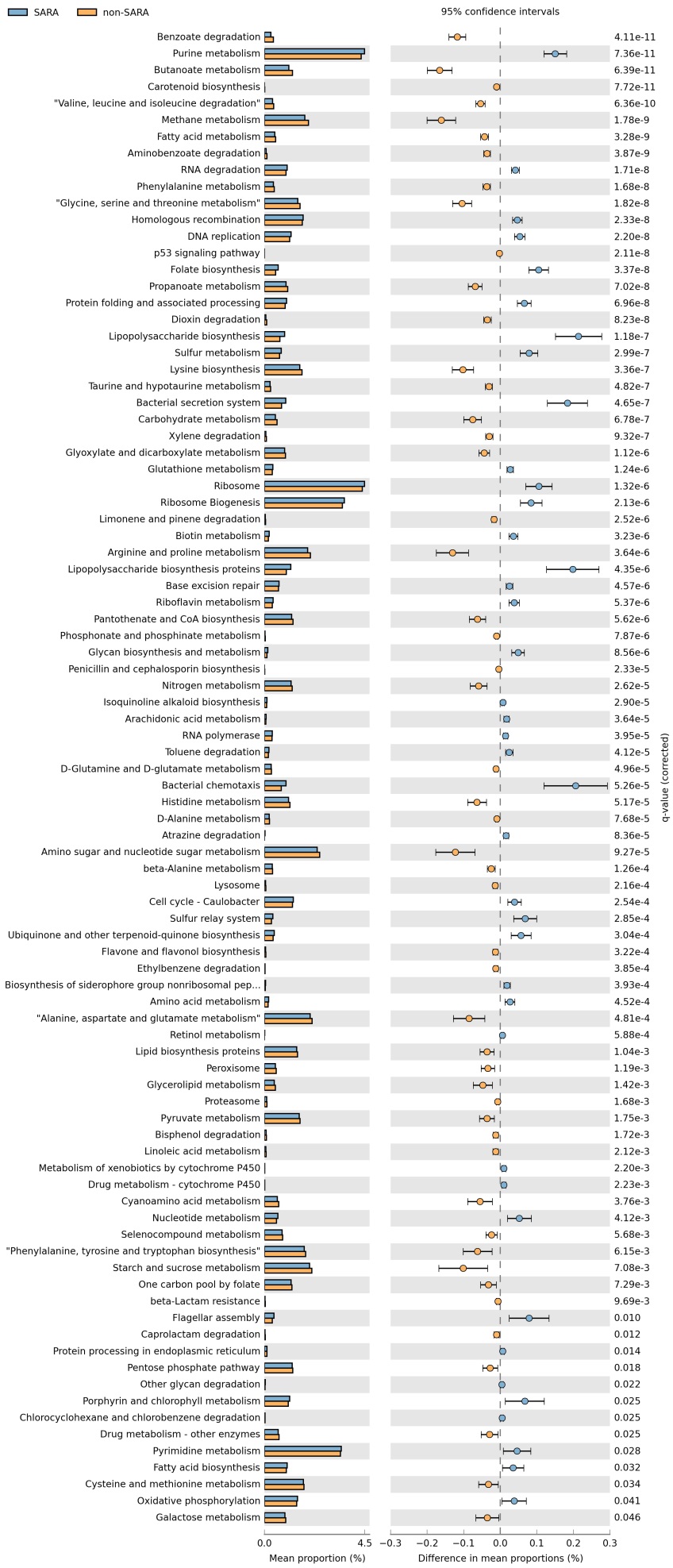

Supplement: Supplementary file 5 — Additional file 5. Differences in predicted microbial metabolic pathways between nonSARA (Pre-SARA1, Post-SARA1 and Post-SARA2) and SARA (SARA1/1, SARA1/2, SARA2/1, SARA2/2) stages in control group. [file 40104_2024_1056_MOESM5_ESM.docx]
