# Supplementary material for: Postbiotics from Saccharomyces cerevisiae fermentation stabilize microbiota in rumen liquid digesta during grain-based subacute ruminal acidosis (SARA) in lactating dairy cows
Source: J Anim Sci Biotechnol. 2024 Aug 1;15:101. doi: 10.1186/s40104-024-01056-x (PMC11293205; doi:10.1186/s40104-024-01056-x)

**Supplementary information**

**Postbiotics from *Saccharomyces cerevisiae* fermentation stabilize microbiota in rumen liquid digesta during grain-based subacute ruminal acidosis (SARA) in lactating dairy cows**

**Additional file 6** Differences in predicted microbial metabolic pathways between nonSARA (Pre-SARA1, Post-SARA1 and Post-SARA2) and SARA (SARA1/1, SARA1/2, SARA2/1, SARA2/2) stages in SCFPb-2X group. Functionalities of rumen liquid microbiome were predicted by CowPi and the results were analyzed by STAMP following log transformation and False Discovery Rate (FDR) correction. Significant differences were considered as *P* < 0.05


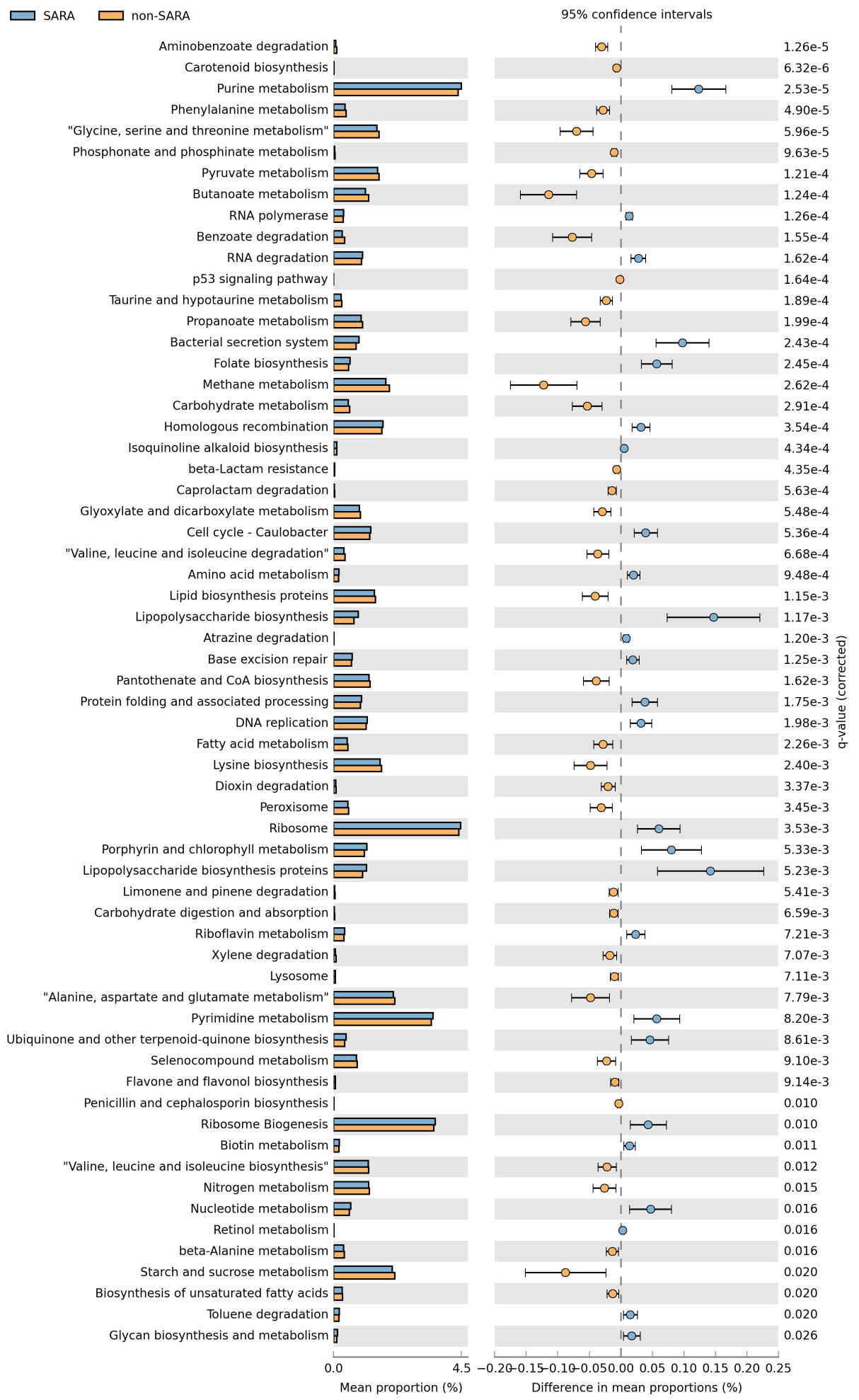

Supplement: Supplementary file 6 — Additional file 6. Differences in predicted microbial metabolic pathways between nonSARA (Pre-SARA1, Post-SARA1 and Post-SARA2) and SARA (SARA1/1, SARA1/2, SARA2/1, SARA2/2) stages in SCFPb-2X group. [file 40104_2024_1056_MOESM6_ESM.docx]
