# Supplementary material for: Postbiotics from Saccharomyces cerevisiae fermentation stabilize microbiota in rumen liquid digesta during grain-based subacute ruminal acidosis (SARA) in lactating dairy cows
Source: J Anim Sci Biotechnol. 2024 Aug 1;15:101. doi: 10.1186/s40104-024-01056-x (PMC11293205; doi:10.1186/s40104-024-01056-x)
Supplement: Supplementary file 7 — Additional file 7. Differences in predicted microbial metabolic pathways between nonSARA (Pre-SARA1, Post-SARA1 and Post-SARA2) and SARA (SARA1/1, SARA1/2, SARA2/1, SARA2/2) stages in SCFPb-1X group. [file 40104_2024_1056_MOESM7_ESM.docx]

**Supplementary information**

**Postbiotics from *Saccharomyces cerevisiae* fermentation stabilize microbiota in rumen liquid digesta during grain-based subacute ruminal acidosis (SARA) in lactating dairy cows**

**Additional file 7** Differences in predicted microbial metabolic pathways between nonSARA (Pre-SARA1, Post-SARA1 and Post-SARA2) and SARA (SARA1/1, SARA1/2, SARA2/1, SARA2/2) stages in SCFPb-1X group. Functionalities of rumen liquid microbiome were predicted by CowPi and the results were analyzed by STAMP following log transformation and False Discovery Rate (FDR) correction. Significant differences were considered as *P* < 0.05

**
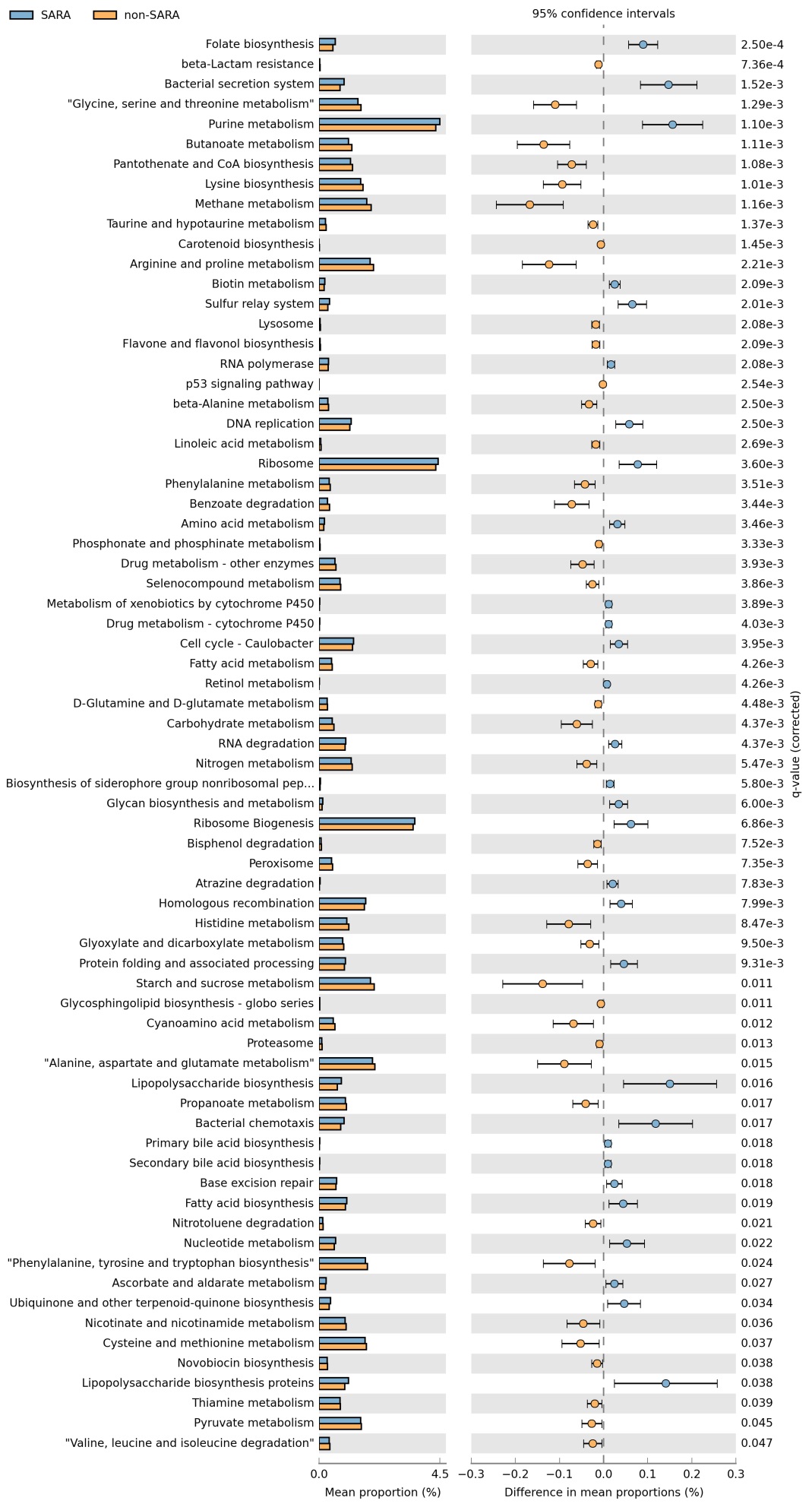
**
